# Genomic characterization and outcome of prosthetic joint infections caused by *Staphylococcus aureus*

**DOI:** 10.1038/s41598-020-62751-z

**Published:** 2020-04-03

**Authors:** Peter Wildeman, Staffan Tevell, Carl Eriksson, Amaya Campillay Lagos, Bo Söderquist, Bianca Stenmark

**Affiliations:** 1 Department of Orthopedics, Faculty of Medicine and Health, Örebro University, Örebro, Sweden; 2 School of Medical Sciences, Faculty of Medicine and Health, Örebro University, Örebro, Sweden; 3 Department of Infectious Diseases, Karlstad, and Centre for Clinical Research, Region Värmland, Karlstad, Sweden; 4 Department of Laboratory Medicine, Faculty of Medicine and Health, Örebro University, Örebro, Sweden

**Keywords:** Outcomes research, Risk factors

## Abstract

*Staphylococcus aureus* is a commensal colonizing the skin and mucous membranes. It can also act as a pathogen, and is the most common microorganism isolated from prosthetic joint infections (PJIs). The aim of this study was to explore the genomic relatedness between commensal and PJI *S. aureus* strains as well as microbial traits and host-related risk factors for treatment failure. Whole-genome sequencing (WGS) was performed on *S. aureus* isolates obtained from PJIs (n = 100) and control isolates from nares (n = 101). Corresponding clinical data for the PJI patients were extracted from medical records. No PJI-specific clusters were found in the WGS phylogeny, and the distribution of the various clonal complexes and prevalence of virulence genes among isolates from PJIs and nares was almost equal. Isolates from patients with treatment success and failure were genetically very similar, while the presence of an antibiotic-resistant phenotype and the use of non-biofilm-active antimicrobial treatment were both associated with failure.In conclusion, commensal and PJI isolates of *S. aureus* in arthroplasty patients were genetically indistinguishable, suggesting that commensal *S. aureus* clones are capable of causing PJIs. Furthermore, no association between genetic traits and outcome could be demonstrated, stressing the importance of patient-related factors in the treatment of *S. aureus* PJIs.

## Introduction

Worldwide, millions of patients undergo arthroplasty surgery every year, with an expected increase in the coming years^1^. *Staphylococcus aureus* is a major cause of both community-acquired and nosocomial infections, and is regularly reported to be the most common pathogen in prosthetic joint infections (PJIs)^2,3^. Risk factors for developing PJIs are related to deficiencies in host defense due to age, obesity, previous surgery, smoking, and immune deficiencies (e.g. rheumatoid arthritis, diabetes mellitus), as well as exogenous factors such as timing and selection of antibiotic prophylaxis, extended operation time, and blood transfusions^4–8^. Implant-sparing curative treatment of acute PJIs, whether occurring early in the postoperative period or later through hematogenous seeding, relies on a regimen of debridement, antibiotics, irrigation, and retention of the prosthesis (DAIR)^9^. This is possible, provided that the infection has not persisted for more than approximately three weeks and that the isolate is susceptible to biofilm-active antibiotic treatment (i.e. rifampin for staphylococcal infections)^10^. However, there are reports suggesting an even shorter time to intervention is needed for a successful treatment^11,12^. The impact of risk factors on outcome of treatment has not been fully explored, but recent reports have proposed new risk scores to better guide the choice of treatment^13–15^.

*S. aureus* is a commensal of the human skin, nares, and mucous membranes, but also a human pathogen due to its invasive capacity. *S. aureus* infections occur among both otherwise-healthy individuals and those with co-morbidities, and nasal carriage has been associated with invasive disease^16^. The pathogenesis of *S. aureus* infection in PJIs involves several critical steps: invasion of host tissues, evasion of the immune system, adhesion to surfaces, and biofilm formation^3,17^. Cell surface components, enzymes, and exotoxins are important virulence factors for invasion, evasion, and propagation^3^. By persisting in biofilm, bacteria evade neutrophil killing and display decreased susceptibility to antibiotics^17,18^, which creates significant challenges in the treatment of PJIs. Previously, *S. aureus* strains isolated from bone and joint infections have been characterized through phenotypic assays and targeted PCRs^19^, but commensal isolates were not included in that study.

The aims of the present study were therefore to characterize a cohort of patients with PJIs caused by *S. aureus*, and to compare the genomes of the corresponding isolates with those of nasal commensals to explore possible associations between microbial traits and outcome of treatment of infection.

## Material and methods

### Ethical approval

The study was approved by the Regional Ethical Review Board of Uppsala, Sweden (ref: 2016/151, 2016/151/1, and 2016/151/2). The collection of nasal bacterial isolates in 1992 was approved by a Chair’s decision of the Regional Ethical Committee in Örebro, Sweden. Informed consent was obtained from all participants in accordance with the Act Concerning the Ethical Review of Research Involving Humans (SFS 2003:460).

### Setting

Patients were included retrospectively between 2004 and 2016 in Region Örebro County and between 2012 and 2016 in Region Värmland County. National Swedish guidelines recommending biofilm-active antimicrobial treatment and DAIR for PJI were introduced in 2004. These two counties together had a recorded population ranging from 550,000 to 574,000 during 2004–2016^20^. They are served by six hospitals, two of which are referral centers with one microbiological department each. During the study period, 14,000 primary and revision hip and knee arthroplasties were performed in Region Örebro (2004–2016), and approximately 4,000 patients underwent surgery in Region Värmland (2012–2016)^21,22^.

National Swedish guidelines recommending DAIR followed by biofilm-active treatment for early postoperative and late acute PJI were introduced 2004. Swedish first-line treatment of PJIs caused by methicillin-susceptible *S. aureus* (MSSA) are intravenous betalactam, in case of a mono-microbial infection, cloxacillin for 7–14 days^23^. Rifampin in combination with a fluoroquinolone is the standard follow-up regimen. Clindamycin, fusidic acid, trimethoprim/sulfamethoxazole, or linezolid are second-line options for rifampin combination therapy. Three months of treatment is recommended. Oral flucloxacillin, for a minimum of six weeks, is the recommended treatment following the first stage of a two-stage procedure in MSSA PJIs.

### Bacterial isolates and patients

All bacterial isolates obtained from PJIs are routinely stored at the microbiological departments, and it was thus possible to cross-reference them with the medical records^24^. All PJIs with a positive culture of *S. aureus* were included in the present study. PJIs were characterized according to the definition by Zimmerli^10^: early postoperative if the infection was manifested within one month of implantation, late acute if the diagnosis was made after an uneventful postoperative period ranging from several weeks to many years after surgery, and chronic if the symptoms had persisted for more than three weeks. A PJI was considered polymicrobial if the culture showed growth of *S. aureus* and one or more additional species. The most common pathogens in polymicrobial infections were coagulase-negative staphylococci (n = 9) followed by streptococci (n = 8), *Enterobacteriales* (n = 5), and *Finegoldia magna* (n = 3). Of the 104 episodes of PJI initially identified, four cases were excluded; two were originally misidentified (one *Staphylococcus argenteus* and one *Staphylococcus lugdunensis*), and two isolates had not been stored at the laboratory. In total, 100 isolates from perioperative tissue biopsies (n = 52), sterile joint aspirates (n = 11), or both (n = 36) were eligible for analysis. In one case of late acute PJI, the *S. aureus* isolate was recovered from blood cultures only. Consequently, 100 cases fulfilled the inclusion criteria and were eligible for further analysis. A retrospective review of the medical records was performed. Co-morbidities, indications for primary surgery, type of prosthetic device, pre-operative laboratory findings, and symptoms at diagnosis of PJI were recorded, as well as the antimicrobial susceptibility testing (AST) report and data on antibiotic treatment. The median follow-up time was 26 months; however, one patient was lost to follow-up due to drug abuse and thus excluded from the outcome analysis. Demographic data are shown in Table 1 and duration of antibiotic treatment in Supplementary Table 1.

**Table 1:** Characteristics of patients with prosthetic joint infections caused by *Staphylococcus aureus*.

Age, years, median (range)	74 (39–92)
**Age**	
<60	18% (18/100)
60–69	19% (19/100)
70–79	34% (34/100)
≥80	29% (29/100)
**Obesity** (BMI > 30 kg/m^2^)	29% (28/97)
**Sex** (female)	50% (50/100)
**Hemoglobin**, g/L, median (range)	123 (82–166)
**ASA** ≥ **3**	51% (51/100)
**Active smoking**	12% (12/100)
**COPD**	9% (9/100)
**Autoimmune disease**	14% (14/100)
**Diabetes**	12% (12/100)
**Immunosuppressive treatment**	13% (13/100)
**Liver cirrhosis**	1% (1/100)
**Malignancy**	2% (2/100)
**Drug abuse**	4% (4/100)
**Prior surgery**	28% (28/100)
**Localization**	
Hip	69% (69/100)
Knee	31% (31/100)
**Indication for surgery**	
Osteoarthritis	62% (62/100)
Rheumatoid arthritis	4% (4/100)
Aseptic loosening	2% (2/100)
Fracture	19% (19/100)
Other	13% (13/100)
**Type of implant**	
Cemented	93% (93/100)
Uncemented	7% (7/100)
**Type of surgery**	
Primary	81% (81/100)
Revision	19% (19/100)
**Classification**	
Early	57% (57/100)
Late acute	25% (25/100)
Chronic	18% (18/100)
**Primary surgical intervention**	
DAIR	83% (83/100)
Two-stage revision	6% (6/100)
One-stage revision	1% (1/100)
Girdlestone	4% (4/100)
No revision	6% (6/100)
**Positive blood culture**	
No	44% (44/100)
Yes	35% (35/100)
Yes (>4 weeks before infection)	6% (6/100)
No sample	15% (15/100)
**Symptoms at diagnosis**	
Wound secretion	61% (61/100)
Pain	79% (79/100)
Fever	54% (54/100)
Redness	62% (62/100)
Sinus tract	20% (20/100)
**C-reactive protein**, mg/mL, median (range)	147 (3–490)
**Blood, leukocyte cell count** > 15 × 10^9^/L	48% (48/100)
**Change of mobile components**	68% (68/100)
**>1 DAIR**	11% (11/100)
**Biofilm-active treatment**	69% (69/100)
**Guideline-compliant treatment**	79.4% (77/97)
**Polymicrobial PJI including** ***S***. ***aureus***	24% (24/100)
**Antibiotic-resistant** ***S***. ***aureus*** (any)	10% (10/100)
**Methicillin-resistant** ***S***. ***aureus***	1% (1/100)

ASA, American Society of Anesthesiologists class; COPD, chronic obstructive pulmonary disease; DAIR, debridement, antibiotics, irrigation and retention of the prosthesis; >1 DAIR, more than one DAIR procedure in the early postoperative phase (<4 weeks), prior surgery, whether there was any earlier surgery in the affected joint e.g. joint replacement, osteotomy, osteosynthesis implants. Guideline-compliant treatment, according to Swedish national guidelines.

Nasal isolates of *S. aureus* were obtained from a separate cohort of anonymized patients awaiting elective arthroplasty surgery at the Department of Orthopedic Surgery, Örebro University Hospital in 1992 (n = 46) and 2017 (n = 55). All isolates were stored in preservation medium (Trypticase soy broth with 0.3% yeast extract and 29% horse serum) at −80 °C.

### Antibiotic susceptibility testing

Antibiotic susceptibility testing was performed by disc diffusion test according to EUCAST guidelines, using version 8.0 (2018) of the EUCAST breakpoint tables for interpretation of MICs and zone diameters^25^. Antibiotic testing was performed in concordance with the Swedish Reference Group for antibiotics; the antibiotics tested were cefoxitin (30 µg), fusidic acid (10 µg), clindamycin (2 µg), erythromycin (15 µg), gentamicin (10 µg), rifampicin (5 µg), trimethoprim-sulfamethoxazole (25 µg), and norfloxacin (10 µg) (all discs from Oxoid, Basingstoke, UK).

### Definitions of outcome

To fulfill the aims of the study, two definitions for outcome were used. First, *treatment success* was intended to describe whether it was possible to achieve cure with implant retention, regardless of infection type or co-infecting pathogens. However, as 24 of the 100 PJI episodes were polymicrobial, and one of the aims was to correlate virulence factors in *S. aureus* to outcome, there was also a need to define *microbiological eradication* of *S. aureus* infection.

Treatment failure was defined as at least one of the following^15^: (a) need for prosthesis removal (one- or two-stage exchange, resection arthroplasty, or amputation), (b) persistent clinical and laboratory signs of infection, (c) need for suppressive antibiotic treatment of any pathogen including *S. aureus*, or d) death during antibiotic treatment when no other obvious explanation was apparent. Neither antibiotic treatment for reasons other than PJI nor use of a second DAIR to control the infection in the early postoperative phase was considered a failure. If none of the above criteria were met, treatment was considered a success regarding overall outcome.

Microbiological eradication was defined as all of the following: (a) no signs of infection and no antibiotic treatment directed at *S. aureus* (regardless of whether the primary intervention required prosthesis removal, i.e. one-stage, two-stage, or resection arthroplasty), (b) no *S. aureus*-associated relapse after completed primary intervention (relapse was still considered a microbiological cure if there was no evidence of remaining *S. aureus* infection in perioperative cultures at re-revision; that is, the relapse was caused by pathogens other than *S. aureu*s), and (c) the patient did not die during PJI treatment.

Outcome was evaluated at the end of follow-up when the patient was either cured of PJI, chronically infected, or dead.

### Whole-genome sequencing and genome analysis

DNA from cultured isolates was automatically extracted using the QIAsymphony DSP Virus/Pathogen kit (Qiagen, Hilden, Germany) according to manufacturer’s instructions, with added RNase treatment and elution in Tris-HCl pH 8.

Whole-genome sequencing was performed with the Nextera XT kit (Illumina Inc, San Diego, CA, USA) on a MiSeq (Illumina) using either v2 2 × 250 bp or v3 2 × 300 paired-end workflow with coverage 50–120×. The reads were trimmed until the average Phred quality was 30 in a window of 20 bases, and *de novo* assembled with version 1.1.04 of Velvet^26^ using optimized k-mer size within version 4.0.2 of SeqSphere+^27^ (Ridom GmbH, Münster, Germany). The whole-genome sequence read files were deposited in the European Nucleotide Archive (ENA) under study accession no. PRJEB33164.

Multilocus sequence typing (MLST) was performed using SeqSphere + (Ridom GmbH), and the sequence types (STs) were subsequently related to clonal complexes (CCs) using eBURST^28^. Species-specific schemes within SeqSphere+ were used to compare the genomes: a core genome multi-locus sequence typing (cgMLST) scheme for comparison of the 1,861 core loci in *S. aureus*, and an accessory typing scheme with 706 accessory loci^29^. The AlereMicroarray schemes were used for comparison of genes involved in virulence (n = 101), resistance (n = 60), and regulation (n = 15)^29^. Loci that were flagged as failed (i.e. found but bearing frameshifts, or a differing consensus sequence, or having a too-low coverage) were considered absent. Phylogenetic trees were constructed in SeqSphere+ using a neighbor-joining tree^30^; missing values were pairwise ignored. The cluster-alert distance was set at a default of 24 allelic differences. The trees were loaded into version 11.0 of CLC Genomics Workbench (Qiagen) for visualization with metadata.

### Genes associated with antibiotic resistance

Presence of the genes *mecA, fusB, fusC, ermA, ermB, ermC, aac-aphD*, and *dfrA* was predicted *in silico* using the resistance scheme for *S. aureus* within SeqSphere + (Ridom GmbH). Presence of the trimethoprim resistance gene *dfrG* was searched *in silico* using previously described primers^31^. Rifampin-resistant isolates were searched for point mutations in the *rpoB* gene as previously described^32^. Isolates resistant to the fluoroquinolone ciprofloxacin were searched for point mutations previously described^33^ in the genes *gyrA*, *gyrB*, *parC*, and *parE*.

### Statistical analysis

Continuous variables with a skew distribution including age, BMI, C-reactive protein, and white blood cell count are presented as median (range). Simpson’s diversity index (DI) was used to evaluate the diversity between PJI isolates and nasal carriage isolates^34^. The DI determines the probability that two randomly picked strains are separated into different typing groups. Categorical data such as sex and gene occurrence in *S. aureu*s are presented as numbers (percentages). The χ^2^ test and Fisher’s exact test were used to compare categorical data between groups and to evaluate the potential influence of categorical variates on the outcome of PJIs (i.e. on treatment outcome, microbiological eradication, and death). Correspondingly, possible associations between covariates on a continuous scale and the outcome of PJIs were evaluated using the Mann-Whitney U test. All covariates with possible association with the outcome of PJIs, defined as a *p-*value < 0.12, were entered into logistic regression models in order to adjust the results for the potential confounders age and sex. All tests were two‐tailed, and statistical significance was defined as *p* ≤ 0.05 or a 95% confidence interval (CI) excluding 1.00 for an odds ratio (OR). Statistical analyses were performed using version 25 of SPSS (IBM Corp., Armonk, NY, 2017).

**Table 2:** Antibiotic susceptibility pattern of *Staphylococcus aureus* in prosthetic joint infection (PJI) and nasal commensal isolates.

	PJI (n = 100)		Nasal (n = 101)	
	Phenotype resistance *(%)	Genotype resistance (%)	Phenotype resistance (%)	Genotype resistance (%)
**Methicillin** (*mecA*)	1 (1.0)	1(1.0)	0 (0.0)	0 (0.0)
**Fusidic acid** (*fusB/C*)	1 (1.0)	1 (1.0)	1 (1.0)	1 (1.0)
**Erythromycin** (*ermA/B/C*)	1 (1.0)	1 (1.0)	1 (1.0)	1 (1.0)
**Clindamycin** (*ermA/B/C*)	2 (2.0)	2 (2.0)	1 (1.0)	1 (1.0)
**Gentamicin** (*aac-aphD*)	0 (0.0)	0 (0.0)	1 (1.0)	1 (1.0)
**Rifampin** (*rpoB*)	3 (3.0)	1 (1.0)	0 (0.0)	0 (0.0)
**Trimethoprim-Sulphamethoxazole** (*dfrA/dfrG*)	0 (0.0)	0 (0.0)	1 (1.0)	0 (0.0)
**Ciprofloxacin** (*gyrA/gyrB/parC/parE*)	6 (6.0)	2 (2.0)	0 (0.0)	0 (0.0)

^*^One PJI isolate was resistant to ciprofloxacin and rifampin, while the rest of the resistant isolates displayed resistance against one agent.

**Table 3:** Risk factors associated with treatment success and failure among patients with *Staphylococcus aureus* prosthetic joint infection.

	Success	Failure	*p*-value^a^	OR for failure (95% CI)^b^
Age, years, median (range)	72.5 (48–91)	75 (39–92)	0.39	
**Age ≥ 80**	22.0% (11/50)	36.7% (18/49)	0.11	1.96 (0.74–5.17)
**Obesity** (BMI > 30 kg/m^2^)	35.4% (17/48)	22.4% (11/49)	0.15	
**Sex** (female)	52.0% (26/50)	49.0% (24/49)	0.76	
**Hemoglobin**, g/L, median (range)	124.5 (82–153)	123.0 (83–166)	0.54	
**ASA ≥ 3**	36.0% (18/50)	63.2% (31/49)	0.007	**3.61 (1.45–9.01)**
**Active smoking**	10.0% (5/50)	10.2% (5/49)	1.00	
**COPD**	8.0% (4/50)	10.2% (5/49)	0.74	
**Autoimmune disease**^**c**^	10.0% (5/50)	22.4% (11/49)	0.02	**5.58 (1.37–22.65)**
**Diabetes**	6.0% (3/50)	16.3% (8/49)	0.12	
**Immunosuppression**	10.0% (5/50)	16.3% (8/49)	0.39	
**Drug abuse**	0.0% (0/50)	6.1% (3/49)	0.12	
**Malignancy**	0.0% (0/50)	4.1% (2/49)	0.24	
**Prior surgery**	22.0% (11/50)	32.7% 16/49)	0.23	
**Localization**				
Hip	68.0% (34/50)	69.4% (34/49)	0.88	
Knee	32.0% (16/50)	30.6% (15/49)		
**Indication for surgery**^**d**^				
Osteoarthritis	72.0% (36/50)	56.5% (26/46)	0.11	Reference
Rheumatoid arthritis	4.0% (2/50)	4.3% (2/46)	0.93	1.47 (0.18–11.11)
Aseptic loosening	4.0% (2/50)	0% (0/49)	0.27	*
Fracture	16.0% (8/50)	21.7% (10/46)	0.47	1.72 (0.55–5.26)
**Type of implant**				
Cemented	94.0% (47/50)	93.9% (46/49)	0.98	
Uncemented	6.0% (3/50)	6.1% (3/49)		
**Type of surgery**				
Primary	84.0% (42/50)	79.6% (39/49)	0.57	
Revision	16.0% (8/50)	20.4% (10/49)		
**Classification**				
Early	78.0% (39/50)	36.7% (18/49)	0.001	**0.02 (0.00–0.19)**
Chronic	0.0% (0/50)	34.7% (17/49)	0.001	Reference
Late acute	22.0% (11/50)	28.6% (14/49)	0.45	**0.07 (0.01–0.63)**
**Positive blood culture**				
No	48.0% (24/50)	40.8% (20/49)	0.47	
Yes	36.0% (18/50)	32.7% (16/49)	0.73	
Yes (>4 weeks before infection)	0.0% (0/50)	12.2% (6/49)	0.12	
No sample	16.0% (8/50)	14.3% (7/49)	0.812	
**Symptoms at diagnosis**				
Wound secretion	70.0% (35/50)	49.0% (24/49)	0.03	**0.40 (0.18–0.93)**
Pain	32.0% (16/50)	87.8% (43/49)	0.02	**3.89 (1.32–11.45)**
Fever	56.0% (28/50)	49.0% (24/49)	0.48	
Redness	66.0% (33/50)	55.1% (27/49)	0.31	
Sinus tract	14.0% (7/50)	22.4% (11/38)	0.31	
**C-reactive protein, high** (>150 mg/L)	50.0% (25/50)	46.9% (23/49)	0.84	
**Blood, leukocyte cell count (>15 × 10**^**9**^**/L)**	20.9% (9/43)	29.5% (14/44)	0.26	
**Change of mobile components**	70.0% (35/50)	65.3% (32/49)	0.69	
**>1 DAIR**	10.0% (5/50)	12.2% (6/49)	0.72	
**Biofilm-active treatment**	90.0%(45/50)	33.8% (23/49)	0.001	**0.09 (0.03–0.28)**
Guideline-compliant treatment ^+^	84.0%(42/50)	73.9% (34/46)	0.22	
**Polymicrobial PJI**	38.0% (19/50)	8.2% (4/49)	0.001	**0.13 (0.04–0.44)**
***S. aureus*** **antibiotic resistance**	0.0% (0/50)	18.3% (9/49)	0.001	

^a^χ^2^-test, Fisher’s exact test, or Mann–Whitney U test.

^b^Covariates with possible associations (p < 0.12) were entered into logistic regression model with adjustments for sex and age. Odds ratios with *p*-values <0.05 are in bold.

^c^Rheumatoid arthritis, systemic lupus erythematosus, sarcoidosis.

^d^Ten patients had other indications.

^*^Too few patients to perform a valid statistical analysis.

^+^Three patients who died before oral treatment stopped were excluded.

**Table 4:** Risk factors for microbiologically persistent *Staphylococcus aureus* prosthetic joint infection.

	Eradicated	Non-eradicated	*p*-value^a^	OR for non-eradication (95% CI)^b^
Age, years, median (range)	73 (46–91)	76.5 (39–92)	0.22	
**Age ≥ 80**	18.6% (12/59)	42.5% (17/40)	0.013	**3.83 (1.44–10.23)**
**Obesity** (BMI > 30 kg/m^2^)	33.3% (19/57)	22.5% (9/40)	0.25	
**Sex** (female)	54.2% (32/59)	45.0% (18/40)	0.37	
**Hemoglobin**, g/L, median (range)	124 (82–153)	123 (83–166)	0.40	
**ASA ≥ 3**	39.0% (23/59)	65.0% (26/40)	0.01	**3.30 (1.30–8.35)**
**Active smoking**	10.2% (6/59)	10.0% (4/40)	1.00	
**COPD**	8.5% (5/59)	10.0% (4/40)	1.00	
**Autoimmune disease**	10.2% (6/59)	20.0% (8/40)	0.17	
**Diabetes**	8.5% (5/59)	15.0% (6/40)	0.34	
**Immunosuppression**^**c**^	11.9% (7/52)	15.0% (6/40)	0.65	
**Drug abuse**	1.7% (1/59)	5.0% (2/40)	0.56	
**Malignancy**	1.7% (1/59)	5.0% (2/40)	0.56	
**Prior surgery**	25.4% (15/44)	30.0% (12/40)	0.62	
**Localization**				
Hip	60.3% (41/68)	39.7% (27/68)	0.83	
Knee	58.1% (18/31)	41.9% (13/31)		
**Indication for surgery**^**d**^				
Osteoarthritis	72.9% (43/ 59)	51.4% (19/37)	0.05	Reference
Rheumatoid arthritis	3.4% (2/57)	5.4% (2/37)	0.64	2.27 (0.29–17.97)
Aseptic loosening	3.4% (2/59)	0.0% (0/39)	0.52	*
Fracture	13.6% (8/59)	27.0% (10/37)	0.10	2.56 (0.84–7.82)
**Type of implant**				
Cemented	94.9% (56/59)	92.5% (3/40)	0.62	
Uncemented	5.1% (3/59)	7.5% (3/40)		
**Type of surgery**				
Primary	83.1% (49/59)	80.% (32/40)	0.70	
Revision	16.9% (10/59)	20.0% (8/40)		
**Classification**				
Early	67.8% (40/59)	42.5% (17/40)	0.02	**0.31 (0.10–0.95)**
Chronic	11.9% (7/59)	25.0% (10/40)	0.19	Reference
Late acute	20.3% (12/59)	32.5% (13/40)	0.23	0.87 (0.25–3.00)
**Positive blood culture**				
No	45.8% (27/59)	42.5% (17/40)	0.75	
Yes	32.2% (19/59)	37.5% (15/40)	0.59	
Yes (>4 weeks before infection)	3.9% (2/59)	10.0% (4/40)	0.18	
No sample	18.6% (11/59)	10.0% (4/40)	0.24	
**Symptoms at diagnosis**				
Wound secretion	66.1% (39/59)	50.0% (20/40)	0.11	0.49 (0.21–1.13)
Pain	71.2% (42/59)	87.5% (35/40)	0.06	3.59 (1.15–11.24)
Fever	54.2% (32/59)	50.0% (20/20)	0.68	
Redness	61.0% (36/59)	60.0% (24/40)	0.92	
Sinus tract	16.9% (10/59)	20.0% (8/40)	0.70	
**C-reactive protein High** (>150 mg/L)	181.9/ 137.9	167.4/156.0	0.86	
**Blood, leukocyte cell count** (>15 × 10^9^/L)	25.5% (1/51)	33.3% (12/36)	0.48	
**Change of mobile components**	74.6% (44/59)	57.5% (23/40)	0.08	0.46 (0.19–1.09)
**>1 DAIR**	8.5% (5/59)	15.0% (6/40)	0.31	
**Biofilm-active treatment**	79.7% (47/59)	47.5% (19/40)	0.004	**0.70 (0.11–0.70)**
**Guideline-compliant treatment**^**+**^	84.7% (50/59)	70.3% (26/37)	0.09	0.42 (0.16–1.12)
**Polymicrobial PJI**	33.9% (20/59)	7.5% (3/40)	0.003	**0.13 (0.04–0.50)**
***S. aureus*** **antibiotic resistance**	1.7% (1/59)	20.0% (8/40)	0.003	**15.99 (1.88–135.91)**

^a^χ^2^-test, Fisher’s exact test, or Mann–Whitney U test.

^b^Covariates with possible associations (p < 0.12) were entered into logistic regression model with adjustments for sex and age. Odds ratios with *p*-values <0.05 are in bold.

^c^Rheumatoid arthritis, systemic lupus erythematosus, sarcoidosis.

^d^Ten patients had other indications.

^*^Too few patients to perform a valid statistical analysis.

^+^Three patients who died before oral treatment stopped were excluded.

## Results

### Comparison between prosthetic joint infection isolates and nasal isolates

**Figure 1:**
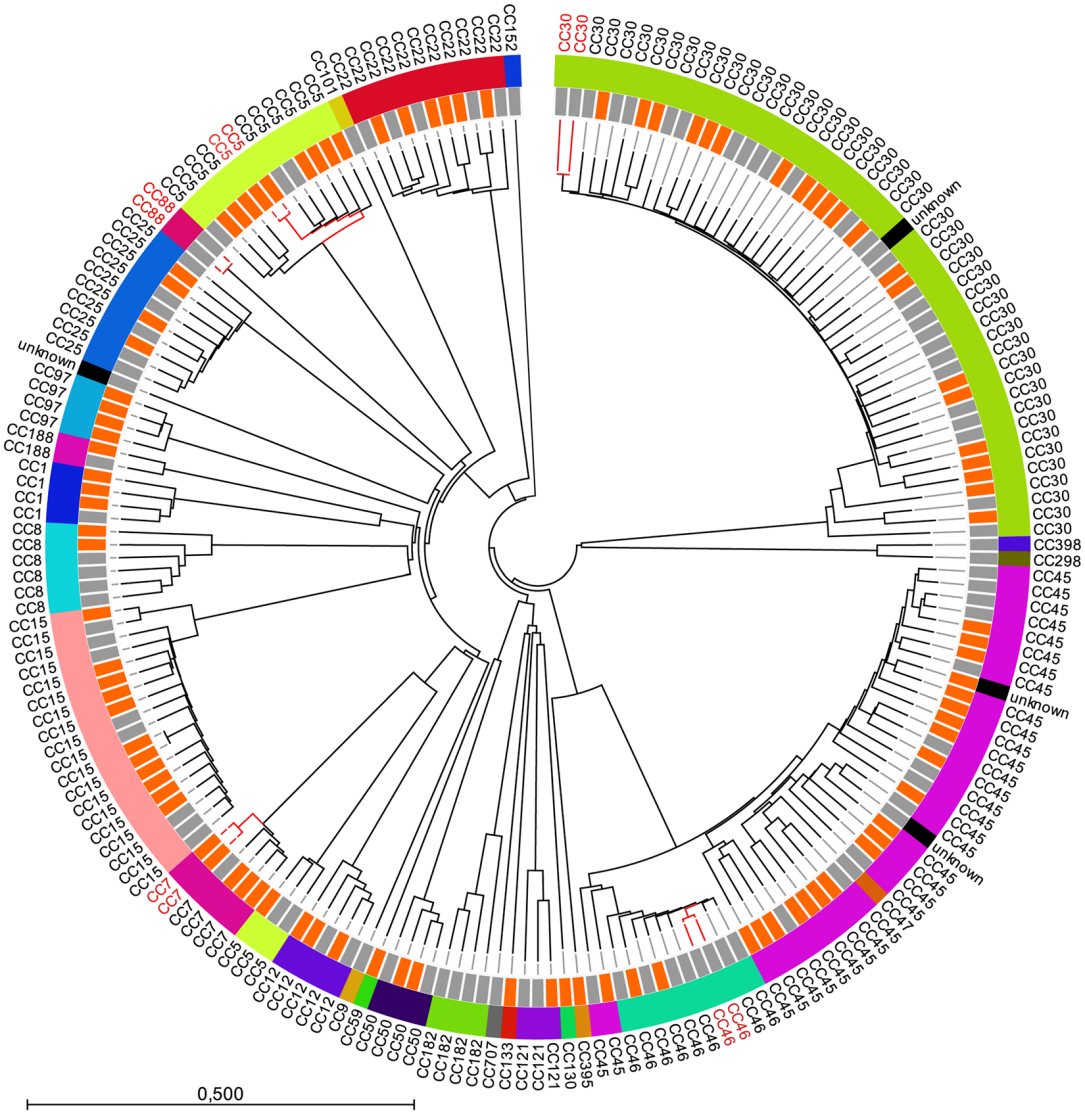
Neighbor-joining tree of core genome multilocus sequence typing (cgMLST) loci (n = 1,861) in *Staphylococcus aureus* isolates (n = 201). The innermost track represents site of collection (prosthetic joint infections in orange and nares in grey) and the outermost track shows clonal complexes (CCs). Five cgMLST complex types (isolates with <25 loci differences) have been marked in red. The scale represents the fraction of core loci differing between the isolates.

cgMLST and MLST-CC showed no PJI-specific clusters, and the distribution among isolates from PJIs and the nasal cohort was almost equal and highly intermingled (Fig. 1). Nineteen different CCs were found among the PJI isolates and 24 different CCs among the nasal isolates (including unknown STs). The three most common CCs in both groups were CC30, CC45, and CC15. On the ST and CC level, Simpson’s diversity index showed equal levels of variance between the two groups (ST: PJI #38 DI 0.94; CI: 0.92–0.96; nares #41 DI 0.93; CI: 0.90–0.96/CC: PJI #19 DI 0.89; CI: 0.86–0.92; nares #23 0.89; CI: 0.85–0.93). There was a high correlation between CCs and cgMLST clusters (Fig. 1). However, three isolates that differed only in two MLST genes, and therefore belonged to the same CC (CC5), were separated by allelic differences in approximately 78% (1,450/1,861) of all cgMLST genes.

Isolates with allelic differences in fewer than 25 loci were clustered into cgMLST complex types; the PJI and nasal isolates were differentiated into 98 different cgMLST complex types each. There were two cgMLST clusters among PJI isolates belonging to CC5 and CC7, with two isolates in each cluster separated by allelic differences in 0 and 10 cgMLST loci, respectively (Fig. 1). The two isolates belonging to CC7 and separated by 10 loci had one single-nucleotide polymorphism (SNP) in each locus, and were from the same patient but from different joints. The first episode was a two-stage revision with eradication of *S. aureus* of the left hip, and the second an early postoperative PJI after primary surgery for osteoarthritis of the right hip; there was more than a year and a half between the two episodes. For the cluster in CC5, there was no epidemiological connection. Another three cgMLST clusters were detected among the nasal isolates but no epidemiological data were available for these isolates.

PJI and nasal isolates did not differ in the prevalence of virulence genes (n = 101) except for *etA*, where seven of the nasal isolates carried genes encoding for this kind of exotoxin compared to none of the PJI isolates (*p* = 0.01), and *lukD*, where 47 of the 100 PJI isolates and 33 of the 100 nasal isolates carried the gene (*p* = 0.04) (Supplementary Table 5) No statistically significant differences in regulatory genes (n = 15) were found between the nasal and the PJI isolates, although *agrII* was found in 13 of the nasal and 26 of the PJI isolates (*p* = 0.06) (Supplementary Table 6).

Methicillin resistance was found in one (1%) PJI isolate and in none of the nasal isolates (Table 2). This MRSA isolate (belonging to ST-10061, CC1) was also resistant to clindamycin and erythromycin. However, this patient was lost to follow-up and excluded from outcome analysis. One nasal commensal was resistant to clindamycin, erythromycin, trimethoprim-sulfamethoxazole, and gentamicin, thus representing the only multi-drug resistant isolate.

**Table 5:** Risk factors for one-year all-cause mortality among patients with *Staphylococcus aureus* prosthetic joint infection (PJI).

	Dead^a^	Alive	*p*-value^b^	OR for death (95% CI)^bc^
**Age**, years, median (range)	82 (66–90)	73 (39–92)	0.001	**1.15 (1.05–1.25)**
**Age ≥ 80**	66.7% (10/15)	22.6% (19/84)	<0.001	**9.97 (2.59–38.28)**
**Obesity** (BMI > 30 kg/m^2^)	26.7% (4/15)	29.3% (24/82)	1.00	
**Sex** (female)	46.7% (7/15)	51.2% (43/84)	0.37	
**Hemoglobin**, g/L, median (range)	103 (83–150)	131 (82–166)	<0.001	**1.06 (1.02–1.11)**
**ASA ≥ 3**	93.3% (14/15)	41.7% (35/84)	<0.001	**12.13 (1.38–106.35)**
**Active smoking**	15.4% (2/13)	9.6% (8/83)	0.62	
**COPD**	6.7% (1/15)	9.5% (8/84)	1.00	
**Autoimmune disease**	13.3% (2/15)	14.3% (12/84)	1.00	
**Diabetes**	13.3% (2/15)	10.7% (9/84)	0.67	
**Immunosuppression**^**d**^	13.3% (2/15)	13.1% (11/84)	1.00	
**Drug abuse**	0.0% (0/15)	3.6% (3/84)	1.00	
**Malignancy**	6.7% (1/15)	2.4% (2/84)	0.39	
**Prior surgery**	20.0% (3/15)	28.6% (24/84)	0.75	
**Localization**				
Hip	80.0% (12/15)	66.7% (56/84)	0.38	
Knee	20.0% (3/15)	33.3% (28/84)		
**Indication for surgery**^**e**^				
Osteoarthritis	25.0% (3/12)	70.2% (59/84)	0.004	Reference
Rheumatoid arthritis	0.0% (0/12)	4.8% (4/84)	1.00	*
Aseptic loosening	0.0% (0/12)	2.4% (2/84)	1.00	*
Fracture	50.0% (6/12)	14.3% (12/84)	0.009	3.80 (0.91–14.64)
**Type of implant**				
Cemented	93.3% (14/15)	94.0% (79/84)	1.00	
Uncemented	6.7% (1/15)	6.0% (5/84)		
**Type of surgery**				
Primary	80.0% (12/15)	71.4% (60/84)	0.75	
Revision	20.0% (3/15)	28.6% (24/84)		
**Classification**				
Early	53.3% (8/15)	58.3% (49/84)	0.78	
Chronic	13.3% (2/15)	17.9% (15/84)	0.73	
Late acute	33.3% (5/15)	23.8% (20/84)	0.52	
**Positive blood culture**				
No	26.7% (4/15)	47.6% (40/84)	0.13	
Yes	46.7% (7/15)	32.1% (27/84)	0.28	
Yes (>4 weeks before infection)	13.3% (2/15)	4.8% (4/84)	0.22	
No sample	13.3% (2/15)	15.5% (13/84)		
**Symptoms at diagnosis**				
Wound secretion	46.7% (7/15)	61.9% (52/84)	0.27	
Pain	86.7% (13/15)	76.2% (64/84)	0.51	
Fever	33.3% (5/15)	56.0% (47/84)	0.11	
Redness	53.3% (8/15)	61.9% (52/84)	0.53	
Sinus tract	20.0% (3/15)	17.8% (15/84)	1.00	
**C-reactive protein, high** (>150 mg/L)	46.7% (7/15)	48.9% (41/84)	1.00	
**Blood, leukocyte cell count** (>15 × 10^9^/L)	35.7% (5/14)	27.4% (20/73)	0.53	
**Change of mobile components**	66.7% (10/15)	67.9% (57/84)	1.00	
**>1 DAIR**	13.3% (2/15)	10.7% (9/84)	0.67	
**Biofilm-active treatment**	26.7% (4/15)	76.2% (64/84)	<0.001	**0.17 (0.03–0.46)**
**Guideline-compliant treatment**^**+**^	75.0% (9/12)	79.8% (67/84)	0.71	
**Polymicrobial PJI**	13.3% (2/15)	17.1% (13/84)	0.51	
***S. aureus*** **antibiotic resistance**	13.3% (2/15)	7.1% (6/84)	0.35	

^a^Dead within 12 months of the PJI diagnosis.

^b^χ^2^-test, Fisher’s exact test, or Mann–Whitney U test.

^c^Covariates with possible associations (p < 0.12) were entered into logistic regression model with adjustments for sex and age. Odds ratios with *p*-values < 0.05 are in bold.

^d^Rheumatoid arthritis, systemic lupus erythematosus, sarcoidosis.

^e^Ten patients had other indications.

^*^Too few patients to perform a valid statistical analysis.

^+^Three patients who died before oral treatment stopped were excluded.

### Patient outcome and genomic traits of *S. aureus*

**Figure 2:**
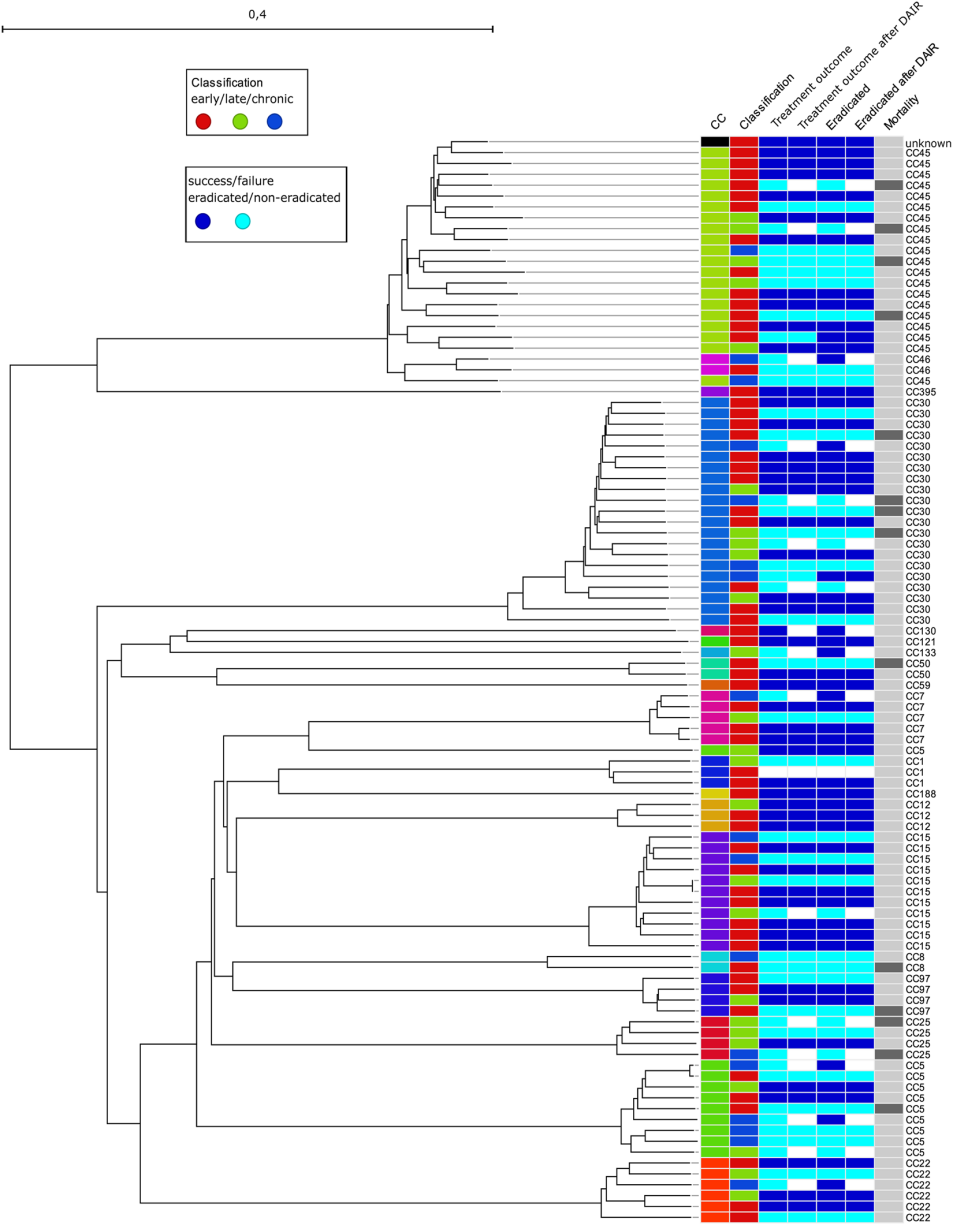
Neighbor-joining tree of core genome multilocus sequence typing (cgMLST) loci (n = 1,861) in *Staphylococcus aureus* isolates from prosthetic joint infections (n = 100). Classification: early postoperative, late acute, and chronic prosthetic joint infection. Eradicated: microbiological eradication of *S. aureus* infection. Mortality: death within 12 months, dark grey. CC: clonal complex. DAIR: debridement, antibiotics, and retention of the prosthesis. The scale represents the fraction of core loci differing between the isolates.

The phylogenetic relationship on the cgMLST level between the different isolates from PJIs in correlation to patient outcome is shown in Fig. 2. Similar clustering was obtained when comparing the core genomes (Fig. 2), accessory genomes, and virulence genes (Fig. S1), which generally followed the MLST-CC groupings. There were no statistically significant differences in treatment outcome (remaining implant and no infection), eradication of the *S. aureus* infection (free of *S. aureus* infection, with or without implant *in situ*), or mortality between the different clusters (data not shown). When the presence of virulence, resistance, and accessory genes was compared between the groups, no statistically significant differences were found except for the capsular polysaccharide (*cap*) genes *cap5H* and *sspP*, which were associated with treatment failure. The *cap8H, cap8I, cap8J and cap8K* genes were associated with treatment success (Supplementary Table 2). *cap8H* and *cap8I* were associated with eradication of *S. aureus* (Supplementary Table 3). When adjusting for the confounders age and sex, the associations for *cap5H, 8H, 8I, 8 J, 8 K* and treatment outcome remained. *cap8H* was still significant for eradication. However, there were no statistically significant associations for treatment outcome (Supplementary Table 2), eradication (Supplementary Table 3), or death (Supplementary Table 4), when a sensitivity analysis for classification was performed.

### Primary surgical and non-surgical intervention for PJI

**Figure 3:**
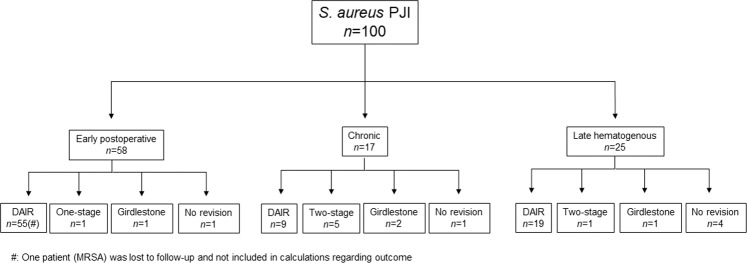
Flowchart of PJI patients regarding classification and primary surgical intervention. (#): One patient (MRSA) was lost to follow-up and not included in the calculations regarding treatment outcome, microbial eradication, and death.

Primary surgical intervention for PJI was DAIR (83%; 82/99). Treatment pathway in terms of classification is given in Fig. 3. One patient was lost to follow-up and excluded from the analysis. DAIR showed a treatment success rate of 60% (49/82) and a microbiological eradication rate of 62% (51/82) regarding *S. aureus* infection after the first intervention (Fig. 2). When the nine chronic cases were removed from the analysis, the treatment success rate increased to 67% (49/73) and the microbiological eradication rate to 68% (50/73). Two-stage exchange was performed in six patients, with 100% eradication rate. Five of these patients were classified as having chronic infections and one as having late acute PJI. The remaining 11 patients with either chronic, early, or late acute PJI had one-stage (n = 1) or resection arthroplasty (n = 4) performed as the first intervention, while six patients were considered too ill for any additional surgery and thus received suppressive antibiotic treatment only. Further analysis of the 17 chronic infections showed that no patient achieved treatment success (Table 3), but seven achieved eradication of *S. aureus* (Table 4). Nine patients had their prosthesis exchanged, three had resection arthroplasty, another three received lifelong suppressive antibiotic treatment, and two died within a year.

### Clinical risk factors for overall treatment failure

Overall treatment failure rate was 49% (49/99) at follow-up. The risk factors for treatment failure are given in Table 3. Factors significantly associated with an increased risk of failure were American Society of Anesthesiologists (ASA) physical status classification ≥3 (OR: 3.61; CI: 1.45–9.01) and autoimmune disease (OR: 5.58; CI: 1.37–22.65). Early postoperative (OR: 0.02; CI: 0.00–0.19) and late acute (OR: 0.07; CI: 0.01–0.63) infections had higher success rate than PJIs classified as chronic infections. Secretion from wounds at the time of diagnosis (OR: 0.40; CI: 0.18–0.93) was associated with success, while pain was associated with failure (OR: 3.89; CI: 1.32–11.45). Patients who received biofilm-active treatment (rifampin ≥ 4 weeks) (OR: 0.09; CI: 0.03–0.28) and patients with polymicrobial infections had a better treatment outcome (OR: 0.13; CI: 0.04–0.44). There was a strong association between antibiotic resistance and failure, with 100% (n = 9) treatment failure among patients infected with a *S. aureus* isolate resistant to at least one agent (*p* = 0.001). Thus, it was not possible to use logistic regression to adjust the observed association between antibiotic resistance and failure for the potential influence of age and sex. No associations between antibiotic resistance and age or sex were observed (*p* = 0.94 and *p* = 1.00). Antibiotic resistance was evenly distributed among the three classification groups (*p* = 0.20).

As classification is an important risk factor for treatment failure, a sensitivity analysis was performed in which all statistically significant associations were adjusted for the impact of the covariate classification (i.e. early, late acute, or chronic). The significant associations between ASA ≥ 3 (OR: 4.92; CI: 1.67–14.38) and age ≥ 80 (OR: 3.36; CI: 1.20–9.43) with treatment failure remained. In addition, biofilm-active treatment (rifampin) showed a significant association with success (OR: 0.09; CI: 0.03–0.32). In contrast, the impacts of autoimmune disease (OR: 3.17; CI: 0.70–14.42), wound secretion (OR: 0.57; CI: 0.16–2.04), pain (OR: 3.88; CI: 0.88–12.92), and polymicrobial infection (OR: 0.28; CI 0.08–1.05) on the risk of treatment failure did not remain significant.

### Clinical risk factors for non-eradication of *S. aureus*

Microbiological eradication of *S. aureus* was not achieved in 40% (40/99) of the patients. The risk factors for non-eradication are given in Table 4. Patients with age ≥ 80 years (OR: 3.83; CI: 1.44–10.23) and ASA ≥ 3 (OR: 3.30; CI: 1.30–8.35) had a higher risk for microbiologically persistent infection. Early postoperative infection had the highest likelihood for eradication (OR: 0.31; CI: 0.10–0.95) compared with chronic infections. Patients with late acute infections (OR: 0.87; CI: 0.25–3.00) were not at higher risk of persistent infection, compared with chronic infections. The covariates pain at diagnosis (OR: 3.59; CI: 1.15–11.24), biofilm-active treatment (OR: 0.70; CI: 0.11–0.70), and polymicrobial infections (OR: 0.13; CI: 0.04–0.50) were all associated with the risk of failed *S. aureus* eradication. A strong association between antibiotic resistance and failure of eradication (OR: 15.99; CI: 1.88–135.91) was observed. A sensitivity analysis in which all significant results were adjusted for the impact of classification revealed that age ≥ 80 (OR: 4.38; CI: 1.57–12.23), ASA ≥ 3 (OR: 3.26; CI: 1.25–8.49), polymicrobial infection (OR: 0.18; CI: 0.04–0.74), biofilm-active treatment (OR: 0.29; CI: 0.11–0.77), and antibiotic resistance (OR: 14.19; CI: 1.62–123.99) were associated with the risk of failure, while pain did not remain significant.

### Clinical risk factors for mortality

All-cause mortality one year after PJI diagnosis was 15% (15/99); risk factors are given in Table 5. Higher age was associated with risk of death (OR: 1.15; CI: 1.05–1.25), especially among those aged ≥ 80 (OR: 9.97; CI: 2.59–38.28). The only co-morbidities associated with an increased risk of one-year mortality were ASA score ≥3 (OR: 12.13; CI: 1.38–106.35) and a low preoperative median hemoglobin level of 103 versus 131 g/L (OR: 1.06; CI: 1.02–1.11). Fracture patients seemed to have a higher risk of mortality than those operated because of osteoarthritis, but the difference did not reach significance (OR: 3.80; CI: 0.91–14.64). There was no significant difference between early (*p* = 0.78), late acute (*p* = 0.52), and chronic PJIs (*p* = 0.73). Patients with S. *aureus* bacteremia (SAB) (n = 27) showed a numerically higher one-year mortality than those with negative blood cultures (n = 40), but the difference was not statistically significant (20.0% vs. 9.1%, *p* = 0.20, Supplementary Table 7). Positive blood cultures were seen in 63.6% (17/25) of patients aged ≥ 80 compared to 33% of patients aged < 60 (*p* = 0.03) (Supplementary Table 7).

## Discussion

*S. aureus* is a common pathogen causing severe morbidity and mortality in patients with PJIs. It displays a wide range of virulence factors involved in adhesion, damage to host cells, and evasion of the immune system^3^, contributing to its pathogenicity. There is limited knowledge about the impact of genomic determinants of the causative agent on patient outcome in PJI. Using whole-genome data, the present study investigated associations between genotypic and phenotypic characteristics and microbiological eradication of infection and success of treatment in PJIs. In addition, genome analysis was used to compare *S. aureus* isolated from PJIs to commensal *S. aureus* isolated from nares. The results showed that the commensal nasal isolates were from the same CCs as the PJI isolates, mainly CC30, CC45, CC15, and CC5. Previous studies have shown conflicting results regarding whether specific lineages of *S. aureus* are related to invasive disease and degree of invasiveness^35–37^. The present commensal nasal isolates displayed the same genetic determinants as the PJI isolates and exhibited a great diversity within the groups. Since there were no differences regarding clonality, we conclude that *S. aureus* PJIs can be caused by multiple CCs, in contrast to *S. epidermidis* where specific lineages (i.e. ST2 and ST215) are more prone to cause PJIs^38^. It could also be hypothesized that the *S. aureus* that causes PJIs originates from the patient’s own microbiota rather than from nosocomial reservoirs. Further evidence linking *S. aureus* carriage to infection comes from the patient who experienced two separate PJI episodes affecting the right and the left hip with an 18-month interval. These isolates had few genomic differences, indicating that the *S. aureus* originated from the patient himself. If this is true, it supports the concept of preoperative nasal eradication in order to decrease the risk of wound infection and subsequent PJI^39,40^.

Biofilm is a complex structure which is central in the pathogenesis of PJI. The *ica*-operon is involved in the production of polysaccharide intercellular adhesin (PIA) and polymeric N‐acetyl‐glucosamine (PNAG), both of which play important roles in biofilm formation. These genes were present in >90% of both the nasal isolates and the PJI isolates (Supplementary Table 5), supporting the hypothesis that commensal isolates are capable of causing biofilm-associated infections. Furthermore, the *agr-*quorum sensing system has been shown to play a central role both in the formation of biofilm and for the pathogenesis of osteomyelitis^3,41–43^, and *agrII* has previously been implied to produce large amounts of biofilm^37^. In the present study, *agrII* was slightly more common among the PJI isolates compared to nares (*p* = 0.06) (Supplementary Table 6).

It is well established that time is of the essence when treating PJIs; mechanical debridement, extensive irrigation of both prosthesis and wound, antibiotic therapy, and exchange of mobile components (i.e. DAIR) are crucial to remove the adhered biofilm, or a complete removal of the prosthesis must be considered. Antibiotic therapy should be effective against *S. aureus* biofilm, specifically rifampin in combination with another biofilm-active drug, preferably a fluoroquinolone to prevent the emergence of rifampin resistance^44–46^. In the present study, the eradication of *S. aureus* was 78% when biofilm-active antibiotics were used but only 38% otherwise (*p* = 0.001), and biofilm-active treatment also produced a successful treatment outcome in 90% versus 38% of cases (*p* = 0.001). Antibiotic resistance was strongly associated with poor outcome, and no patient with *S. aureus* expressing resistance to any of the tested antibiotics achieved microbiological eradication. Rifampin and fluoroquinolone resistance were the most common, which is of great concern as these are the best documented biofilm-active antibiotic combination for *S. aureus* PJIs after DAIR^10,47^. Thus, if the infection is caused by an antibiotic-resistant *S. aureus*, removal of the prosthesis and later re-implantation (two-stage exchange) should be considered as primary intervention, given that eradication in this study was only achieved if the implant was removed. There is currently no consensus regarding how long after presentation of infection DAIR remains a useful option^10–12^. If the infection is allowed to progress for more than 3 weeks (i.e. chronic infection), DAIR is more likely to fail. This result is to be expected, since a mature biofilm is probably formed within 3 weeks. In the present study all these cases failed; no patient with a chronic PJI had an overall successful treatment outcome, and microbiological *S. aureus* eradication was only achieved following two-stage revision or resection arthroplasty. Some of the patients with late acute onset were classified as chronic PJI because of delay in diagnosis and treatment. In the light of this poor outcome for chronic PJIs, it is imperative not to delay diagnosis of PJIs and to be vigilant for new symptoms from a well-functioning joint prosthesis in the setting of SAB, as the reported PJI rate following SAB with onset in the community has been reported to be 30–40%^48^.

Wound secretion was associated with early PJIs, thus making it easier to diagnose and to administer a timely treatment. In addition to constituting early postoperative infections, these polymicrobial infections may also represent more fulminant infections that are diagnosed and managed earlier, explaining why there was a higher degree of eradication of *S. aureus* in this group. Early postoperative infections have a better outcome than the late acute and chronic PJIs^5,15^. Pain is more difficult to interpret, but is a symptom frequently associated with late acute and chronic PJIs.

Patient characteristics have an impact on the outcome of treatment, as previously reported^13,15^ and as confirmed in the present findings. Higher ASA score, age ≥ 80, and fracture as indication for operation were associated with non-eradication.

Despite the great efforts made to provide effective treatment for *S. aureus* PJIs, it has proven to be associated with high mortality rates^49^. Previous reports of PJI-related one-year mortality have ranged from 4% to 8%^50–52^. The one-year mortality rate in this present cohort was 15%, highlighting that *S. aureus* infections are serious complications of prosthetic joint surgery.

One strength of this study is the low number of missing cases, since we applied two separate approaches to identify patients with PJIs due to *S. aureus*; both by diagnosis code and personal identification numbers, and through the databases of the microbiology departments. Moreover, only one patient was lost to follow-up. However, there are also limitations, including the retrospective cohort design. The nasal isolates were not collected from the patients who subsequently experienced a PJI, but from patients planned for elective orthopedic surgery. Furthermore, without high-quality closed genomes it is possible that some genes that were considered as absent were actually present, but were not detected due to low sequencing coverage. In addition, this was an exploratory study, and in order to maximize the sensitivity of our approach (with a trade-off in specificity), no correction for multiple testing was performed. There were differences in several parameters that could not be confirmed statistically; this could be because of limited number of patients in certain groups, suggesting that these differences might reach significance in a larger cohort.

In conclusion, commensal and PJI isolates of *S. aureus* clones are genetically indistinguishable, indicating that commensal isolates cause PJIs. Eradication of infection and treatment success are both predominately dependent on duration of symptoms, time to surgical intervention, and the presence of resistant phenotypes of *S. aureus*. When the *S. aureus* harbors genotypic or phenotypic characteristics for antimicrobial resistance, especially against biofilm-active antibiotics, extensive surgical intervention is needed to achieve a successful treatment. Thus, in these cases, complete removal of the prosthesis and all its components, performed as one- or two-stage exchange, should be considered as primary intervention to achieve eradication of infection and a successful outcome for the patient.

## Supplementary information

Supplementary information.

Supplementary information2.
