# Supplementary material for: Genomic characterization and outcome of prosthetic joint infections caused by Staphylococcus aureus
Source: Sci Rep. 2020 Apr 3;10:5938. doi: 10.1038/s41598-020-62751-z (PMC7125104; doi:10.1038/s41598-020-62751-z)
Supplement: Supplementary file 2 — Supplementary information2. [file 41598_2020_62751_MOESM2_ESM.docx]

**Genomic characterization and outcome of prosthetic joint infections caused by *Staphylococcus aureus***

**P. Wildeman^1, 2^, S. Tevell, ^2, 3^ C. Eriksson^2^, A. Campillay Lagos^4^, B. Söderquist^2,4^ and B. Stenmark^4^**

1) Department of Orthopedics, Faculty of Medicine and Health, Örebro University, Örebro, Sweden

2) School of Medical Sciences, Faculty of Medicine and Health, Örebro University, Örebro, Sweden

3) Department of Infectious Diseases, Karlstad, and Centre for Clinical Research, Region Värmland, Karlstad, Sweden

4) Department of Laboratory Medicine, Faculty of Medicine and Health, Örebro University, Örebro, Sweden

**Corresponding author**:
Peter Wildeman
Department of Orthopedics
Örebro University Hospital
SE-70185 Örebro
Sweden
Telephone: +46196021000
[peter.wildeman@regionorebrolan.se](mailto:peter.wildeman@regionorebrolan.se)

**Supplementary figures**

**
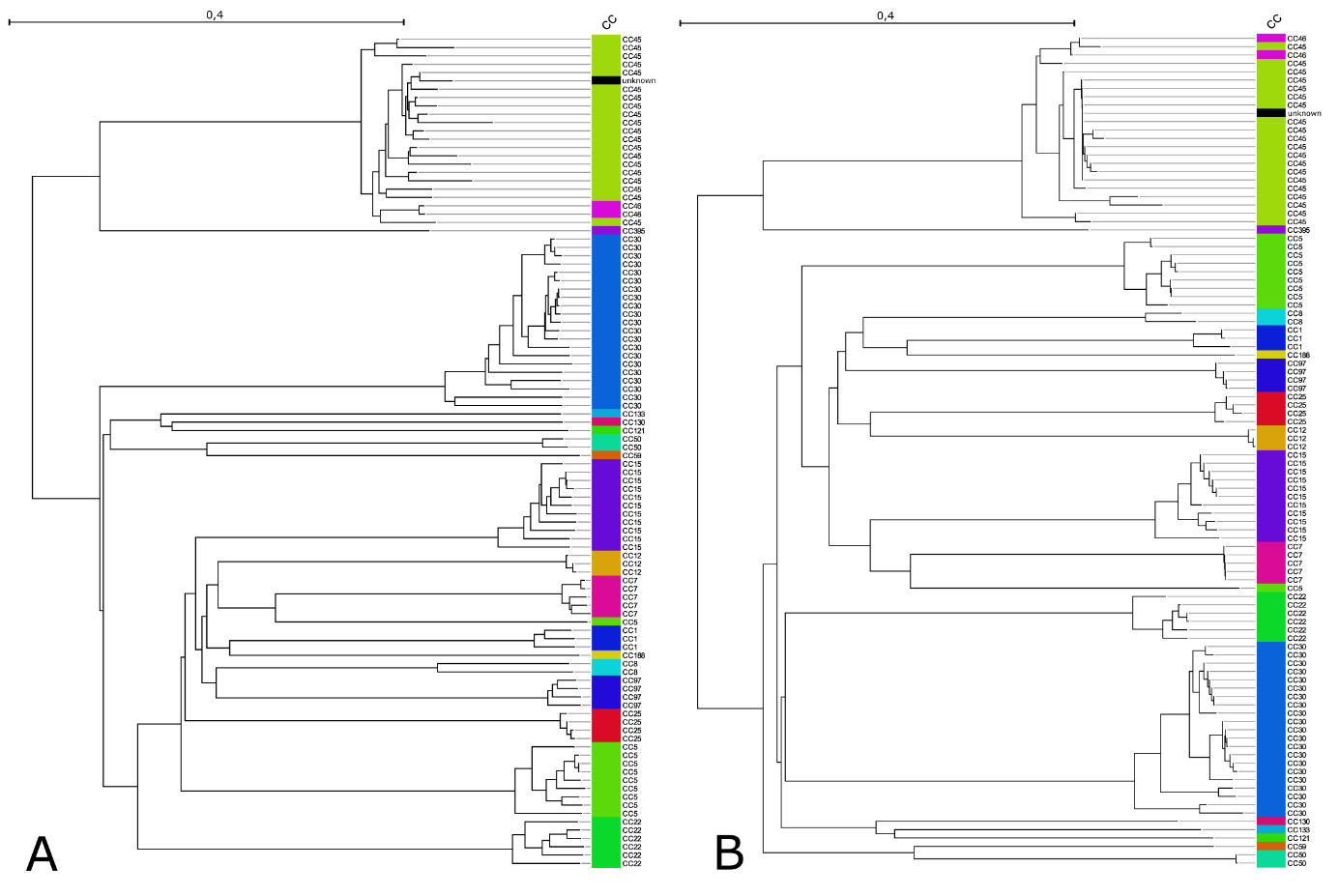
**

**Supplementary figure 1.** Neighbor-joining tree of *Staphylococcus aureus.* A) accessory loci (n=706) and B) virulence loci (n=101) in isolates from prosthetic joint infections (n=100). The scale bar represents % of A) accessory loci and B) virulence loci differing between the isolates.





**Supplementary figure 2.** Distribution of sequence types of *Staphylococcus aureus* among isolates from prosthetic joint infections (n=100) and nares (n=101).

**Supplementary tables**

**Supplementary table 1.** Duration of antibiotic treatment in relation to primary surgical intervention*.

*Numbers are given as mean (minimum-maximum; standard deviation)

(a) Data from only one patient

(b) No data, oral long term suppression treatment

| **Primary surgical intervention** | **Intravenous (days)** | **Oral (months)** | **Total duration (months)** |
| --- | --- | --- | --- |
| All patients (n=100) | 16.2 (1.0-91.0; 12.9) | 3.5 (0.3-9.0; 1.7) | 4.0 (1.5-9.8; 1.7) |
| DAIR (n=83) | 16.2 (1.0-91.0; 12.9) | 3.4 (0.3-8.5; 1.6) | 3.9 (1.5-8.5; 1.5) |
| One-stage exchange (n=1) | 19 (a) | 5 (a) | 5.8 (a) |
| Two-stage exchange (n=6) | 14.5 (7.0-28.0; 8.4) | 4.6 (1.0-9.0; 3.2) | 5.1 (1.5-9.8; 3.3) |
| Resection arthroplasty or arthrodesis (n=4) | 22.5 (3.0-60.0; 25.6) | 1.5 (0.8-2.3; 1.1) | 2.1 (1.5-2.7; 0.6) |
| No revision (n=6) | 13.8 (7.0-28.0; 7.7) | (b) |  |

**Supplementary table 2.** Presence and absence of virulence genes in treatment failure and success in *S. aureus* prosthetic joint infections.

* χ^2^-test or Fisher’s exact test as appropriate.

^2^Covariates with possible associations (*p* <0.12) were entered into logistic regression model with adjustments for sex and age. Odds ratios with *p*-values <0.05 are in bold letters.

^3^Not possible to make regression calculations.

|  |  | **Presence in** | |  |  |
| --- | --- | --- | --- | --- | --- |
| **Virulence genes** | **Gene product** | Success(n=50) | Failure (n=49) | ***p*-value*** | **OR for failure (95% CI)^2^** |
| *ORF-CM14* | Putative enterotoxin | 5 | 0 | 0.06 | ^3^ |
| *arcA* | Arginine deiminase | 0 | 0 | 1.00 |  |
| *arcB* | Ornithine carbamoyltransferase | 0 | 0 | 1.00 |  |
| *arcC* | Carbamate kinase | 0 | 0 | 1.00 |  |
| *arcD* | Arginine/ornithine antiporter | 1 | 0 | 1.00 |  |
| *aur* | Aureolysin | 48 | 49 | 1.00 |  |
| *bap* | Biofilm-associated surface protein | 0 | 0 | 1.00 |  |
| *cap1H* | Capsular polysaccharide 1 | 0 | 0 | 1.00 |  |
| *cap1J* | Capsular polysaccharide 1 | 0 | 0 | 1.00 |  |
| *cap1K* | Capsular polysaccharide 1 | 0 | 0 | 1.00 |  |
| *cap5H* | Capsular polysaccharide 5 | 7 | 17 | **0.02** | **2.83 (1.06-7.60)** |
| *cap5J* | Capsular polysaccharide 5 | 7 | 16 | **0.03** | 2.55 (0.95-6.87) |
| *cap5K* | Capsular polysaccharide 5 | 7 | 16 | **0.03** | 2.55 (0.95-6.87) |
| *cap8H* | Capsular polysaccharide 8 | 36 | 23 | **0.01** | **0.38 (0.17-0.87)** |
| *cap8I* | Capsular polysaccharide 8 | 41 | 31 | **0.02** | **0.38 (0.15- 0.97)** |
| *cap8J* | Capsular polysaccharide 8 | 42 | 32 | **0.02** | **0.35 (0.13-0.95)** |
| *cap8K* | Capsular polysaccharide 8 | 42 | 32 | **0.02** | **0.32 (0.13-0.81)** |
| *chp* | Chemotaxis inhibitory protein CHIPS | 34 | 38 | 0.36 |  |
| *ebpS* | Elastin-binding protein | 47 | 47 | 1.00 |  |
| *edinA* | Epidermal cell differentiation inhibitor A | 0 | 0 | 1.00 |  |
| *edinB* | Epidermal Cell differentiation inhibitor B | 1 | 3 | 0.62 |  |
| *edinC* | Epidermal cell differentiation inhibitor C | 1 | 0 | 1.00 |  |
| *eno* | Enolase | 48 | 49 | 1.00 |  |
| *etA* | Exfoliative toxin A | 0 | 0 | 1.00 |  |
| *etB* | Exfoliative toxin B | 0 | 0 | 1.00 |  |
| *etD* | Exfoliative toxin D | 1 | 1 | 1.00 |  |
| *fib* | Fibrinogen-binding protein | 44 | 42 | 0.54 |  |
| *hl* | Putative hemolysin | 49 | 48 | 1.00 |  |
| *hlIII* | Hemolysin III | 48 | 48 | 1.00 |  |
| *hla* | α-toxin | 46 | 47 | 1.00 |  |
| *hlb-intact* | Beta hemolysin | 4 | 1 | 0.36 |  |
| *hlgA* | Hemolysin gamma subunit | 48 | 46 | 0.62 |  |
| *hlgB* | Hemolysin gamma subunit | 49 | 49 | 1.00 |  |
| *hlgC* | Hemolysin gamma subunit | 49 | 49 | 1.00 |  |
| *icaA* | Intercellular adhesion protein A | 48 | 46 | 0.62 |  |
| *icaC* | Intercellular adhesion protein C | 47 | 44 | 0.44 |  |
| *icaD* | Intercellular adhesion protein D | 49 | 47 | 0.50 |  |
| *indicator-clfA* | Clumping factor A | 48 | 49 | 1.00 |  |
| *indicator-clfB* | Clumping factor B | 48 | 45 | 0.36 |  |
| *indicator-cna* | Collagen binding adhesin | 32 | 27 | 0.30 |  |
| *indicator-ebh* | Extracellular matrix-binding protein homologue | 49 | 49 | 1.00 |  |
| *indicator-fnbA* | Fibronectin-binding protein A | 47 | 48 | 1.00 |  |
| *indicator-fnbB* | Fibronectin-binding protein B | 37 | 37 | 1.00 |  |
| *indicator-hysA* | Hyaluronate lyase | 48 | 48 | 1.00 |  |
| *indicator-map* | Extracellular adhesive protein | 48 | 49 | 1.00 |  |
| *indicator-sasG* | *S. aureus* surface protein G | 17 | 19 | 0.68 |  |
| *indicator-sdrC* | SD-repeats-protein C (Serine-Aspartic acid) | 49 | 49 | 1.00 |  |
| *indicator-sdrD* | SD-repeats-protein D (Serine-Aspartic acid) | 37 | 40 | 0.46 |  |
| *indicator-sdrE* | SD-repeats-protein E (Serine-Aspartic acid) | 35 | 39 | 0.35 |  |
| *indicator-vwb* | Von Willebrand factor-binding protein | 49 | 49 | 1.00 |  |
| *isaB* | Immunodominant antigen B | 49 | 49 | 1.00 |  |
| *isdA* | Iron-regulated surface protein A | 49 | 47 | 0.50 |  |
| *lukD* | Leukocidin D component | 22 | 23 | 0.84 |  |
| *lukE* | Leukocidin E component | 20 | 19 | 0.84 |  |
| *lukF-PV* | Panton-Valentine leukotoxin | 49 | 49 | 1.00 |  |
| *lukF-PV83* | Panton-Valentine leukotoxin | 49 | 49 | 1.00 |  |
| *lukM* | Leukocidin | 49 | 49 | 1.00 |  |
| *lukS-PV* | Panton-Valentine leukotoxin | 1 | 0 | 1.00 |  |
| *lukX* | Putative leukocidin | 48 | 49 | 1.00 |  |
| *lukY* | Putative leukocidin | 47 | 49 | 0.50 |  |
| *sak* | Staphylokinase | 36 | 40 | 0.33 |  |
| *scn* | Staphylococcal complement inhibitor SCIN | 42 | 41 | 0.78 |  |
| *sea-sep* | Staphylococcal enterotoxin A | 11 | 11 | 1.00 |  |
| *seb* | Staphylococcal enterotoxin B | 4 | 0 | 0.12 |  |
| *sec* | Staphylococcal enterotoxin C | 9 | 7 | 0.59 |  |
| *sed* | Staphylococcal enterotoxin D | 0 | 1 | 1.00 |  |
| *see* | Staphylococcal enterotoxin E | 49 | 49 | 1.00 |  |
| *seg* | Staphylococcal enterotoxin G | 23 | 24 | 1.00 |  |
| *seh* | Staphylococcal enterotoxin H | 1 | 1 | 1.00 |  |
| *sei* | Staphylococcal enterotoxin I | 24 | 23 | 0.84 |  |
| *sej* | Staphylococcal enterotoxin J | 0 | 1 | 1.00 |  |
| *sek* | Staphylococcal enterotoxin K | 1 | 1 | 1.00 |  |
| *sel* | Staphylococcal enterotoxin L | 9 | 8 | 1.00 |  |
| *sem* | Staphylococcal enterotoxin M | 25 | 27 | 0.69 |  |
| *sen* | Staphylococcal enterotoxin N | 19 | 14 | 0.29 |  |
| *seo* | Staphylococcal enterotoxin O | 21 | 23 | 0.69 |  |
| *seq* | Staphylococcal enterotoxin Q | 1 | 1 | 1.00 |  |
| *ser* | Staphylococcal enterotoxin R | 0 | 1 | 1.00 |  |
| *setB1* | *setB1* | 49 | 49 | 1.00 |  |
| *setB2* | *setB2* | 48 | 45 | 0.36 |  |
| *setB3* | *setB3* | 47 | 49 | 0.50 |  |
| *setC* | Staphylococcal exotoxin-like protein | 27 | 26 | 1.00 |  |
| *seu* | Staphylococcal enterotoxin U | 11 | 15 | 0.36 |  |
| *splA* | Serine protease A | 22 | 20 | 0.68 |  |
| *splB* | Serine protease B | 22 | 23 | 0.84 |  |
| *splE* | Serine protease E | 28 | 26 | 0.69 |  |
| *ssl01* | *ssl01* | 45 | 45 | 1.00 |  |
| *ssl02* | *ssl02* | 48 | 48 | 1.00 |  |
| *ssl03* | *ssl03* | 44 | 42 | 0.54 |  |
| *ssl04* | *ssl04* | 37 | 36 | 1.00 |  |
| *ssl05* | *ssl05* | 49 | 49 | 1.00 |  |
| *ssl06* | *ssl06* | 11 | 10 | 1.00 |  |
| *ssl07* | *ssl07* | 48 | 46 | 0.62 |  |
| *ssl08* | *ssl08* | 23 | 23 | 1.00 |  |
| *ssl09* | *ssl09* | 47 | 48 | 1.00 |  |
| *ssl10* | *ssl10* | 48 | 47 | 1.00 |  |
| *ssl11* | *ssl11* | 34 | 31 | 0.52 |  |
| *sspA* | Glutamyl endopeptidase | 48 | 46 | 0.62 |  |
| *sspB* | Staphopain B | 49 | 49 | 1.00 |  |
| *sspP* | Staphopain A | 50 | 44 | **0.03** | ^3^ |
| *tst1* | Toxic shock syndrome toxin (TSST)-1 | 9 | 11 | 0.62 |  |

**Supplementary table 3.** Presence and absence of virulence genes in eradicated and non-eradicated *S. aureus* prosthetic joint infection.

* χ^2^-test or Fisher’s exact test as appropriate.

^2^Covariates with possible associations (*p* <0.12) were entered into logistic regression model with adjustments for sex and age. Odds ratios with *p*-values <0.05 are in bold letters.

^3^Not possible to make logistic regression calculations.

|  |  | **Presence in** | |  |  |
| --- | --- | --- | --- | --- | --- |
| **Virulence genes** | **Gene product** | Eradicated (n=59) | Non-eradicated (n=40) | ***p*-value*** | **OR for non-eradiation (95% CI)^2^** |
| *ORF-CM14* | Putative enterotoxin | 5 | 0 | 0.08 | ^3^ |
| *arcA* | Arginine deiminase | 0 | 0 | 1.00 |  |
| *arcB* | Ornithine carbamoyltransferase | 0 | 0 | 1.00 |  |
| *arcC* | Carbamate kinase | 0 | 0 | 1.00 |  |
| *arcD* | Arginine/ornithine antiporter | 0 | 0 | 1.00 |  |
| *aur* | Aureolysin | 59 | 40 | 1.00 |  |
| *bap* | Biofilm-associated surface protein | 0 | 0 | 1.00 |  |
| *cap1H* | Capsular polysaccharide 1 | 0 | 0 | 1.00 |  |
| *cap1J* | Capsular polysaccharide 1 | 0 | 0 | 1.00 |  |
| *cap1K* | Capsular polysaccharide 1 | 0 | 0 | 1.00 |  |
| *cap5H* | Capsular polysaccharide 5 | 11 | 14 | 0.07 | 2.30 (0.88-6.00) |
| *cap5J* | Capsular polysaccharide 5 | 11 | 13 | 0.11 | 2.01 (0.77-5.27) |
| *cap5K* | Capsular polysaccharide 5 | 11 | 13 | 0.11 | 2.01 (0.77-5.27) |
| *cap8H* | capsular polysaccharide 8 | 40 | 18 | **0.02** | **0.39 (0.17-0.90)** |
| *cap8I* | Capsular polysaccharide 8 | 47 | 25 | 0.06 | 0.44 (0.18-1.10) |
| *cap8J* | Capsular polysaccharide 8 | 48 | 26 | 0.07 | 0.44 (0.17-1.13) |
| *cap8K* | Capsular polysaccharide 8 | 46 | 24 | **0.05** | 0.44 (0.18-1.08) |
| *chp* | Chemotaxis inhibitory protein CHIPS | 41 | 32 | 0.24 |  |
| *ebpS* | Elastin-binding protein | 55 | 40 | 0.15 |  |
| *edinA* | Epidermal cell differentiation inhibitor A | 0 | 0 | 1.00 |  |
| *edinB* | Epidermal Cell differentiation inhibitor B | 2 | 3 | 0.39 |  |
| *edinC* | Epidermal cell differentiation inhibitor C | 0 | 0 | 1.00 |  |
| *eno* | Enolase | 59 | 40 | 1.00 |  |
| *etA* | Exfoliative toxin A | 0 | 0 | 1.00 |  |
| *etB* | Exfoliative toxin B | 0 | 0 | 1.00 |  |
| *etD* | Exfoliative toxin D | 0 | 1 | 0.40 |  |
| *fib* | Fibrinogen-binding protein | 52 | 35 | 1.00 |  |
| *hl* | Putative hemolysin | 59 | 39 | 0.40 |  |
| *hlIII* | Hemolysin III | 59 | 39 | 0.40 |  |
| *hla* | α-toxin | 56 | 38 | 1.00 |  |
| *hlb-intact* | Beta hemolysin | 4 | 0 | 0.15 |  |
| *hlgA* | Hemolysin gamma subunit | 58 | 37 | 0.30 |  |
| *hlgB* | Hemolysin gamma subunit | 59 | 40 | 1.00 |  |
| *hlgC* | Hemolysin gamma subunit | 59 | 40 | 1.00 |  |
| *icaA* | Intercellular adhesion protein A | 58 | 37 | 0.30 |  |
| *icaC* | Intercellular adhesion protein C | 57 | 35 | 0.12 |  |
| *icaD* | Intercellular adhesion protein D | 59 | 38 | 1.00 |  |
| *indicator-clfA* | Clumping factor A | 1 | 0 | 1.00 |  |
| *indicator-clfB* | Clumping factor B | 58 | 36 | 0.16 |  |
| *indicator-cna* | Collagen binding adhesin | 36 | 22 | 0.55 |  |
| *indicator-ebh* | Extracellular matrix-binding protein homologue | 59 | 40 | 1.00 |  |
| *indicator-fnbA* | Fibronectin-binding protein A | 57 | 39 | 1.00 |  |
| *indicator-fnbB* | Fibronectin-binding protein B | 45 | 30 | 0.89 |  |
| *indicator-hysA* | Hyaluronate lyase | 58 | 39 | 1.00 |  |
| *indicator-map* | Extracellular adhesive protein | 20 | 16 | 0.54 |  |
| *indicator-sasG* | *S. aureus* surface protein G | 59 | 40 | 1.00 |  |
| *indicator-sdrC* | SD-repeats-protein C (Serine-Aspartic acid) | 59 | 40 | 1.00 |  |
| *indicator-sdrD* | SD-repeats-protein D (Serine-Aspartic acid) | 47 | 31 | 0.80 |  |
| *indicator-sdrE* | SD-repeats-protein E (Serine-Aspartic acid) | 44 | 31 | 0.74 |  |
| *indicator-vwb* | Von Willebrand factor-binding protein | 59 | 40 | 1.00 |  |
| *isaB* | Immunodominant antigen B | 59 | 40 | 1.00 |  |
| *isdA* | Iron-regulated surface protein A | 59 | 38 | 0.16 |  |
| *lukD* | Leukocidin D component | 27 | 19 | 0.87 |  |
| *lukE* | Leukocidin E component | 25 | 16 | 0.84 |  |
| *lukF-PV* | Panton-Valentine leukotoxin | 0 | 0 | 1.00 |  |
| *lukF-PV83* | Panton-Valentine leukotoxin | 0 | 0 | 1.00 |  |
| *lukM* | Leukocidin | 0 | 0 | 1.00 |  |
| *lukS-PV* | Panton-Valentine leukotoxin | 0 | 0 | 1.00 |  |
| *lukX* | Putative leukocidin | 58 | 40 | 1.00 |  |
| *lukY* | Putative leukocidin | 58 | 40 | 1.00 |  |
| *sak* | Staphylokinase | 44 | 33 | 0.35 |  |
| *scn* | Staphylococcal complement inhibitor SCIN | 49 | 35 | 0.55 |  |
| *sea-sep* | Staphylococcal enterotoxin A | 14 | 8 | 0.66 |  |
| *seb* | Staphylococcal enterotoxin B | 4 | 0 | 0.15 |  |
| *sec* | Staphylococcal enterotoxin C | 10 | 6 | 0.80 |  |
| *sed* | Staphylococcal enterotoxin D | 0 | 1 | 0.40 |  |
| *see* | Staphylococcal enterotoxin E | 0 | 0 | 1.00 |  |
| *seg* | Staphylococcal enterotoxin G | 29 | 19 | 0.87 |  |
| *seh* | Staphylococcal enterotoxin H | 1 | 1 | 1.00 |  |
| *sei* | Staphylococcal enterotoxin I | 27 | 20 | 0.68 |  |
| *sej* | Staphylococcal enterotoxin J | 0 | 1 | 0.40 |  |
| *sek* | Staphylococcal enterotoxin K | 1 | 1 | 1.00 |  |
| *sel* | Staphylococcal enterotoxin L | 10 | 7 | 0.94 |  |
| *sem* | Staphylococcal enterotoxin M | 31 | 22 | 0.81 |  |
| *sen* | Staphylococcal enterotoxin N | 20 | 14 | 0.91 |  |
| *seo* | Staphylococcal enterotoxin O | 25 | 20 | 0.46 |  |
| *seq* | Staphylococcal enterotoxin Q | 1 | 1 | 1.00 |  |
| *ser* | Staphylococcal enterotoxin R | 0 | 1 | 0.40 |  |
| *setB1* | *setB1* | 59 | 40 | 1.00 |  |
| *setB2* | *setB2* | 58 | 36 | 0.16 |  |
| *setB3* | *setB3* | 57 | 40 | 0.51 |  |
| *setC* | Staphylococcal exotoxin-like protein | 33 | 21 | 0.74 |  |
| *seu* | Staphylococcal enterotoxin U | 15 | 12 | 0.62 |  |
| *splA* | Serine protease A | 26 | 17 | 0.88 |  |
| *splB* | Serine protease B | 27 | 19 | 0.87 |  |
| *splE* | Serine protease E | 31 | 23 | 0.63 |  |
| *ssl01* | *ssl01* | 54 | 37 | 1.00 |  |
| *ssl02* | *ssl02* | 58 | 39 | 1.00 |  |
| *ssl03* | *ssl03* | 53 | 34 | 0.54 |  |
| *ssl04* | *ssl04* | 43 | 30 | 0.81 |  |
| *ssl05* | *ssl05* | 59 | 40 | 1.00 |  |
| *ssl06* | *ssl06* | 13 | 8 | 1.00 |  |
| *ssl07* | *ssl07* | 57 | 38 | 1.00 |  |
| *ssl08* | *ssl08* | 28 | 19 | 1.00 |  |
| *ssl09* | *ssl09* | 57 | 39 | 1.00 |  |
| *ssl10* | *ssl10* | 58 | 38 | 0.56 |  |
| *ssl11* | *ssl11* | 40 | 25 | 0.59 |  |
| *sspA* | Glutamyl endopeptidase | 57 | 38 | 1.00 |  |
| *sspB* | Staphopain B | 59 | 40 | 1.00 |  |
| *sspP* | Staphopain A | 58 | 36 | 0.16 |  |
| *tst1* | Toxic shock syndrome toxin (TSST)-1 | 12 | 8 | 1.00 |  |
|  | | | | |  |

**Supplementary table 4.** Presence and absence of virulence genes among dead or alive in *S. aureus* prosthetic joint infections.

* χ^2^-test or Fisher’s exact test as appropriate.

^2^Covariates with possible associations (*p* <0.12) were entered into logistic regression model with adjustments for sex and age. Odds ratios with *p*-values <0.05 are in bold letters.

|  |  | **Presence in** | |  |  |
| --- | --- | --- | --- | --- | --- |
| **Virulence genes** | **Gene product** | Dead (n=15) | Alive (n=84) | ***p*-value*** | **OR for death (95% CI)^2^** |
| *ORF-CM14* | Putative enterotoxin | 0 | 5 | 0.43 |  |
| *arcA* | Arginine deiminase | 0 | 0 | 1.00 |  |
| *arcB* | Ornithine carbamoyltransferase | 0 | 0 | 1.00 |  |
| *arcC* | Carbamate kinase | 0 | 0 | 1.00 |  |
| *arcD* | Arginine/ornithine antiporter | 0 | 0 | 1.00 |  |
| *aur* | Aureolysin | 15 | 84 | 1.00 |  |
| *bap* | Biofilm-associated surface protein | 0 | 0 | 1.00 |  |
| *cap1H* | Capsular polysaccharide 1 | 0 | 0 | 1.00 |  |
| *cap1J* | Capsular polysaccharide 1 | 0 | 0 | 1.00 |  |
| *cap1K* | Capsular polysaccharide 1 | 0 | 0 | 1.00 |  |
| *cap5H* | Capsular polysaccharide 5 | 5 | 20 | 0.52 |  |
| *cap5J* | Capsular polysaccharide 5 | 4 | 20 | 0.75 |  |
| *cap5K* | Capsular polysaccharide 5 | 4 | 20 | 0.75 |  |
| *cap8H* | capsular polysaccharide 8 | 9 | 49 | 0.90 |  |
| *cap8I* | Capsular polysaccharide 8 | 10 | 62 | 0.55 |  |
| *cap8J* | Capsular polysaccharide 8 | 10 | 64 | 0.52 |  |
| *cap8K* | Capsular polysaccharide 8 | 10 | 60 | 0.76 |  |
| *chp* | Chemotaxis inhibitory protein CHIPS | 11 | 62 | 1.00 |  |
| *ebpS* | Elastin-binding protein | 15 | 80 | 1.00 |  |
| *edinA* | Epidermal cell differentiation inhibitor A | 0 | 0 | 1.00 |  |
| *edinB* | Epidermal Cell differentiation inhibitor B | 2 | 3 | 0.16 |  |
| *edinC* | Epidermal cell differentiation inhibitor C | 0 | 0 | 1.00 |  |
| *eno* | Enolase | 15 | 84 | 1.00 |  |
| *etA* | Exfoliative toxin A | 0 | 0 | 1.00 |  |
| *etB* | Exfoliative toxin B | 0 | 0 | 1.00 |  |
| *etD* | Exfoliative toxin D | 1 | 0 | 0.15 |  |
| *fib* | Fibrinogen-binding protein | 15 | 72 | 0.20 |  |
| *hl* | Putative hemolysin | 14 | 84 | 0.15 |  |
| *hlIII* | Hemolysin III | 14 | 84 | 0.15 |  |
| *hla* | α-toxin | 15 | 79 | 1.00 |  |
| *hlb-intact* | Beta hemolysin | 0 | 4 | 1.00 |  |
| *hlgA* | Hemolysin gamma subunit | 15 | 84 | 1.00 |  |
| *hlgB* | Hemolysin gamma subunit | 15 | 84 | 1.00 |  |
| *hlgC* | Hemolysin gamma subunit | 15 | 84 | 1.00 |  |
| *icaA* | Intercellular adhesion protein A | 14 | 81 | 0.49 |  |
| *icaC* | Intercellular adhesion protein C | 14 | 78 | 1.00 |  |
| *icaD* | Intercellular adhesion protein D | 15 | 82 | 1.00 |  |
| *indicator-clfA* | Clumping factor A | 15 | 83 | 1.00 |  |
| *indicator-clfB* | Clumping factor B | 13 | 81 | 0.16 |  |
| *indicator-cna* | Collagen binding adhesin | 9 | 49 | 1.00 |  |
| *indicator-ebh* | Extracellular matrix-binding protein homologue | 15 | 84 | 1.00 |  |
| *indicator-fnbA* | Fibronectin-binding protein A | 15 | 81 | 1.00 |  |
| *indicator-fnbB* | Fibronectin-binding protein B | 11 | 64 | 0.75 |  |
| *indicator-hysA* | Hyaluronate lyase | 14 | 83 | 0.28 |  |
| *indicator-map* | Extracellular adhesive protein | 15 | 83 | 1.00 |  |
| *indicator-sasG* | *S. aureus* surface protein G | 3 | 33 | 0.24 |  |
| *indicator-sdrC* | SD-repeats-protein C (Serine-Aspartic acid) | 15 | 84 | 1.00 |  |
| *indicator-sdrD* | SD-repeats-protein D (Serine-Aspartic acid) | 11 | 67 | 1.00 |  |
| *indicator-sdrE* | SD-repeats-protein E (Serine-Aspartic acid) | 11 | 64 | 0.75 |  |
| *indicator-vwb* | Von Willebrand factor-binding protein | 15 | 84 | 1.00 |  |
| *isaB* | Immunodominant antigen B | 15 | 84 | 1.00 |  |
| *isdA* | Iron-regulated surface protein A | 13 | 84 | 1.00 |  |
| *lukD* | Leukocidin D component | 7 | 39 | 1.00 |  |
| *lukE* | Leukocidin E component | 5 | 36 | 0.58 |  |
| *lukF-PV* | Panton-Valentine leukotoxin | 0 | 0 | 1.00 |  |
| *lukF-PV83* | Panton-Valentine leukotoxin | 0 | 0 | 1.00 |  |
| *lukM* | Leukocidin | 0 | 0 | 1.00 |  |
| *lukS-PV* | Panton-Valentine leukotoxin | 0 | 0 | 1.00 |  |
| *lukX* | Putative leukocidin | 15 | 83 | 1.00 |  |
| *lukY* | Putative leukocidin | 15 | 83 | 1.00 |  |
| *sak* | Staphylokinase | 13 | 64 | 0.51 |  |
| *scn* | Staphylococcal complement inhibitor SCIN | 13 | 71 | 1.00 |  |
| *sea-sep* | Staphylococcal enterotoxin A | 3 | 9 | 1.00 |  |
| *seb* | Staphylococcal enterotoxin B | 0 | 4 | 1.00 |  |
| *sec* | Staphylococcal enterotoxin C | 3 | 13 | 0.71 |  |
| *sed* | Staphylococcal enterotoxin D | 0 | 1 | 1.00 |  |
| *see* | Staphylococcal enterotoxin E | 0 | 0 | 1.00 |  |
| *seg* | Staphylococcal enterotoxin G | 8 | 40 | 0.68 |  |
| *seh* | Staphylococcal enterotoxin H | 0 | 2 | 1.00 |  |
| *sei* | Staphylococcal enterotoxin I | 9 | 38 | 0.29 |  |
| *sej* | Staphylococcal enterotoxin J | 0 | 1 | 1.00 |  |
| *sek* | Staphylococcal enterotoxin K | 0 | 2 | 1.00 |  |
| *sel* | Staphylococcal enterotoxin L | 4 | 13 | 0.28 |  |
| *sem* | Staphylococcal enterotoxin M | 8 | 45 | 0.99 |  |
| *sen* | Staphylococcal enterotoxin N | 7 | 27 | 0.28 |  |
| *seo* | Staphylococcal enterotoxin O | 9 | 36 | 0.22 |  |
| *seq* | Staphylococcal enterotoxin Q | 0 | 2 | 1.00 |  |
| *ser* | Staphylococcal enterotoxin R | 0 | 1 | 1.00 |  |
| *setB1* | *setB1* | 15 | 84 | 1.00 |  |
| *setB2* | *setB2* | 12 | 82 | 0.16 |  |
| *setB3* | *setB3* | 15 | 82 | 1.00 |  |
| *setC* | Staphylococcal exotoxin-like protein | 7 | 47 | 0.51 |  |
| *seu* | Staphylococcal enterotoxin U | 6 | 21 | 0.23 |  |
| *splA* | Serine protease A | 5 | 28 | 0.57 |  |
| *splB* | Serine protease B | 7 | 39 | 0.99 |  |
| *splE* | Serine protease E | 10 | 44 | 0.40 |  |
| *ssl01* | *ssl01* | 12 | 79 | 0.10 |  |
| *ssl02* | *ssl02* | 15 | 82 | 1.00 |  |
| *ssl03* | *ssl03* | 13 | 74 | 1.00 |  |
| *ssl04* | *ssl04* | 10 | 63 | 0.53 |  |
| *ssl05* | *ssl05* | 15 | 84 | 1.00 |  |
| *ssl06* | *ssl06* | 2 | 19 | 0.52 |  |
| *ssl07* | *ssl07* | 15 | 80 | 1.00 |  |
| *ssl08* | *ssl08* | 7 | 40 | 0.95 |  |
| *ssl09* | *ssl09* | 14 | 82 | 0.39 |  |
| *ssl10* | *ssl10* | 15 | 81 | 1.00 |  |
| *ssl11* | *ssl11* | 8 | 57 | 0.28 |  |
| *sspA* | Glutamyl endopeptidase | 14 | 81 | 0.49 |  |
| *sspB* | Staphopain B | 15 | 84 | 1.00 |  |
| *sspP* | Staphopain A | 14 | 80 | 0.57 |  |
| *tst1* | Toxic shock syndrome toxin (TSST)-1 | 2 | 18 | 0.73 |  |
|  | | | | |  |

**Supplementary table 5.** Presence and absence of virulence genes in nasal and prosthetic joint infection *S. aureus* isolates.

* χ^2^-test or Fisher’s exact test as appropriate.

|  |  | **Presence in** | |  |
| --- | --- | --- | --- | --- |
| **Virulence genes** | **Gene product** | Nasal carriage (n=101) | PJI  (n=100) | ***p*-value*** |
| *ORF-CM14* | Putative enterotoxin | 5 | 4 | 0.75 |
| *arcA* | Arginine deiminase | 0 | 0 | 1.00 |
| *arcB* | Ornithine carbamoyltransferase | 0 | 0 | 1.00 |
| *arcC* | Carbamate kinase | 0 | 0 | 1.00 |
| *arcD* | Arginine/ornithine antiporter | 1 | 0 | 0.50 |
| *aur* | Aureolysin | 99 | 100 | 1.00 |
| *bap* | Biofilm-associated surface protein | 0 | 0 | 1.00 |
| *cap1H* | Capsular polysaccharide 1 | 0 | 0 | 1.00 |
| *cap1J* | Capsular polysaccharide 1 | 0 | 0 | 1.00 |
| *cap1K* | Capsular polysaccharide 1 | 0 | 0 | 1.00 |
| *cap5H* | Capsular polysaccharide 5 | 27 | 25 | 0.78 |
| *cap5J* | Capsular polysaccharide 5 | 25 | 24 | 1.00 |
| *cap5K* | Capsular polysaccharide 5 | 23 | 24 | 0.84 |
| *cap8H* | capsular polysaccharide 8 | 60 | 60 | 0.93 |
| *cap8I* | Capsular polysaccharide 8 | 73 | 66 | 0.24 |
| *cap8J* | Capsular polysaccharide 8 | 70 | 75 | 0.37 |
| *cap8K* | Capsular polysaccharide 8 | 71 | 69 | 0.76 |
| *chp* | Chemotaxis inhibitory protein CHIPS | 73 | 72 | 0.79 |
| *ebpS* | Elastin-binding protein | 100 | 95 | 0.12 |
| *edinA* | Epidermal cell differentiation inhibitor A | 0 | 0 | 1.00 |
| *edinB* | Epidermal Cell differentiation inhibitor B | 7 | 5 | 0.56 |
| *edinC* | Epidermal cell differentiation inhibitor C | 0 | 1 | 1.00 |
| *eno* | Enolase | 100 | 99 | 1.00 |
| *etA* | Exfoliative toxin A | 7 | 0 | **0.01** |
| *etB* | Exfoliative toxin B | 0 | 0 | 1.00 |
| *etD* | Exfoliative toxin D | 1 | 2 | 0.62 |
| *fib* | Fibrinogen-binding protein | 88 | 88 | 0.85 |
| *hl* | Putative hemolysin | 99 | 99 | 1.00 |
| *hlIII* | Hemolysin III | 101 | 98 | 0.25 |
| *hla* | α-toxin | 96 | 95 | 1.00 |
| *hlb-intact* | Beta hemolysin | 8 | 5 | 0.40 |
| *hlgA* | Hemolysin gamma subunit | 96 | 96 | 1.00 |
| *hlgB* | Hemolysin gamma subunit | 100 | 100 | 1.00 |
| *hlgC* | Hemolysin gamma subunit | 97 | 100 | 0.12 |
| *icaA* | Intercellular adhesion protein A | 97 | 96 | 1.00 |
| *icaC* | Intercellular adhesion protein C | 95 | 93 | 0.78 |
| *icaD* | Intercellular adhesion protein D | 100 | 98 | 0.62 |
| *indicator-clfA* | Clumping factor A | 101 | 99 | 0.50 |
| *indicator-clfB* | Clumping factor B | 95 | 95 | 0.77 |
| *indicator-cna* | Collagen binding adhesin | 72 | 59 | 0.07 |
| *indicator-ebh* | Extracellular matrix-binding protein homologue | 101 | 100 | 1.00 |
| *indicator-fnbA* | Fibronectin-binding protein A | 99 | 97 | 0.68 |
| *indicator-fnbB* | Fibronectin-binding protein B | 65 | 76 | 0.07 |
| *indicator-hysA* | Hyaluronate lyase | 97 | 98 | 0.41 |
| *indicator-map* | Extracellular adhesive protein | 95 | 99 | 0.12 |
| *indicator-sasG* | *S. aureus* surface protein G | 28 | 37 | 0.16 |
| *indicator-sdrC* | SD-repeats-protein C (Serine-Aspartic acid) | 99 | 100 | 0.50 |
| *indicator-sdrD* | SD-repeats-protein D (Serine-Aspartic acid) | 83 | 79 | 0.60 |
| *indicator-sdrE* | SD-repeats-protein E (Serine-Aspartic acid) | 82 | 76 | 0.39 |
| *indicator-vwb* | Von Willebrand factor-binding protein | 99 | 100 | 0.50 |
| *isaB* | Immunodominant antigen B | 101 | 100 | 1.00 |
| *isdA* | Iron-regulated surface protein A | 98 | 97 | 1.00 |
| *lukD* | Leukocidin D component | 33 | 47 | **0.04** |
| *lukE* | Leukocidin E component | 31 | 42 | 0.10 |
| *lukF-PV* | Panton-Valentine leukotoxin | 1 | 0 | 1.00 |
| *lukF-PV83* | Panton-Valentine leukotoxin | 0 | 0 | 1.00 |
| *lukM* | Leukocidin | 0 | 0 | 1.00 |
| *lukS-PV* | Panton-Valentine leukotoxin | 1 | 0 | 1.00 |
| *lukX* | Putative leukocidin | 100 | 99 | 1.00 |
| *lukY* | Putative leukocidin | 100 | 99 | 1.00 |
| *sak* | Staphylokinase | 82 | 78 | 0.58 |
| *scn* | Staphylococcal complement inhibitor SCIN | 85 | 85 | 0.87 |
| *sea-sep* | Staphylococcal enterotoxin A | 25 | 22 | 0.65 |
| *seb* | Staphylococcal enterotoxin B | 4 | 4 | 1.00 |
| *sec* | Staphylococcal enterotoxin C | 10 | 16 | 0.20 |
| *sed* | Staphylococcal enterotoxin D | 4 | 1 | 0.37 |
| *see* | Staphylococcal enterotoxin E | 0 | 0 | 1.00 |
| *seg* | Staphylococcal enterotoxin G | 55 | 48 | 0.40 |
| *seh* | Staphylococcal enterotoxin H | 5 | 3 | 0.48 |
| *sei* | Staphylococcal enterotoxin I | 48 | 47 | 0.94 |
| *sej* | Staphylococcal enterotoxin J | 3 | 1 | 0.62 |
| *sek* | Staphylococcal enterotoxin K | 2 | 2 | 1.00 |
| *sel* | Staphylococcal enterotoxin L | 11 | 17 | 0.21 |
| *sem* | Staphylococcal enterotoxin M | 61 | 53 | 0.32 |
| *sen* | Staphylococcal enterotoxin N | 43 | 34 | 0.25 |
| *seo* | Staphylococcal enterotoxin O | 48 | 45 | 0.72 |
| *seq* | Staphylococcal enterotoxin Q | 1 | 2 | 0.62 |
| *ser* | Staphylococcal enterotoxin R | 4 | 1 | 0.37 |
| *setB1* | *setB1* | 94 | 100 | **0.01** |
| *setB2* | *setB2* | 96 | 95 | 1.00 |
| *setB3* | *setB3* | 94 | 98 | 0.17 |
| *setC* | Staphylococcal exotoxin-like protein | 47 | 55 | 0.23 |
| *seu* | Staphylococcal enterotoxin U | 23 | 27 | 0.52 |
| *splA* | Serine protease A | 32 | 44 | 0.07 |
| *splB* | Serine protease B | 36 | 47 | 0.10 |
| *splE* | Serine protease E | 44 | 54 | 0.14 |
| *ssl01* | *ssl01* | 95 | 92 | 0.57 |
| *ssl02* | *ssl02* | 101 | 98 | 0.25 |
| *ssl03* | *ssl03* | 89 | 88 | 0.98 |
| *ssl04* | *ssl04* | 80 | 74 | 0.38 |
| *ssl05* | *ssl05* | 99 | 100 | 0.50 |
| *ssl06* | *ssl06* | 20 | 22 | 0.73 |
| *ssl07* | *ssl07* | 90 | 96 | 0.06 |
| *ssl08* | *ssl08* | 41 | 48 | 0.32 |
| *ssl09* | *ssl09* | 99 | 97 | 0.68 |
| *ssl10* | *ssl10* | 99 | 97 | 0.68 |
| *ssl11* | *ssl11* | 62 | 66 | 0.50 |
| *sspA* | Glutamyl endopeptidase | 92 | 96 | 0.16 |
| *sspB* | Staphopain B | 100 | 100 | 1.00 |
| *sspP* | Staphopain A | 90 | 95 | 0.12 |
| *tst1* | Toxic shock syndrome toxin (TSST)-1 | 25 | 20 | 0.42 |

**Supplementary table 6.** Presence and absence of regulatory genes in nasal and prosthetic joint infection *S. aureus* isolates.

* X^2^-test or Fisher’s exact test when appropriate

| **Regulatory gene** | **Nasal carriage (n=101)** | **PJI (n=100)** | ***p*-value*** |
| --- | --- | --- | --- |
| *agr* grp I | 48% (48) | 48% (48) | 0.95 |
| *agr* grp II | 13% (13) | 23% (23) | 0.06 |
| *agr* grp III | 30 %(30) | 25% (25) | 0.46 |
| *agr* grp IV | 4 % (4) | 3% (3) | 1.00 |
| *hld* | 91% (91) | 91% (91) | 0.83 |
| *saeS* | 101% (101) | 100% (100) | 1.00 |
| *sarA* | 98 % (98) | 99% (99) | 0.62 |
|  | | | |

**Supplementary table 7.** Frequency of blood cultures stratified by age group and all-cause one-year mortality.

*Growth of *S. aureus* in blood cultures taken more than four weeks before PJI diagnosis.

Yr. Year

|  | Result of blood culture | | | | All-cause mortality | |
| --- | --- | --- | --- | --- | --- | --- |
|  | No Growth | Positive at diagnosis | Positive,  Prior* | None taken | Alive after 1 yr. | Dead within 1 yr. |
| Age | *n (%)* | *n (%)* | *n (%)* | *n (%)* | *n (%)* | *n (%)* |
| All (*n*=100) | 44 (44.0) | 35 (35.0) | 6 (6.0) | 15 (15.0) | 85 (85.0) | 15 (15.0) |
| <60 (*n*=18*)* | 12 (66.7) | 6 (33.3) | 0 (0.0) | 0 (0.0) | 18 (100.0) | 0(0.0) |
| 60-69 (*n*=19) | 7 (36.8) | 7 (36.8) | 1 (5.3) | 4 (21.1) | 18 (94.7) | 1 (5.3) |
| 70-79 (*n*=34) | 17 (50.0) | 8 (23.5) | 2 (5.9) | 7 (20.6) | 30 (88.2) | 4 (11.8) |
| >80 (*n*=29) | 8 (27.6) | 14 (48.3) | 3 (10.3) | 4 (13.8) | 19 (65.5) | 10 (34.5) |
| Alive After 1 yr. | 40 (90.9) | 28 (80.0) | 4 (66.7) | 13 (85.0) | - | - |
| Dead Within 1 yr. | 4 (9.1) | 7 (20.0) | 2 (33.3) | 2 (13.3) | - | - |
